# Ecological Momentary Assessment of Weight-Related Behaviors in the Home Environment of Children From Low-Income and Racially and Ethnically Diverse Households: Development and Usability Study

**DOI:** 10.2196/30525

**Published:** 2021-12-01

**Authors:** Amanda Trofholz, Allan Tate, Mark Janowiec, Angela Fertig, Katie Loth, Junia N de Brito, Jerica Berge

**Affiliations:** 1 Humphrey School of Public Affairs University of Minnestoa Minneapolis, MN United States; 2 College of Public Health University of Georgia Athens, GA United States

**Keywords:** methods, ecological momentary assessment, weight-related behaviors, racially and ethnically diverse, children, mobile phone

## Abstract

**Background:**

Ecological momentary assessment (EMA) is an innovative tool for capturing in-the-moment health behaviors as people go about their daily lives. EMA is an ideal tool to measure weight-related behaviors, such as parental feeding practices, stress, and dietary intake, as these occur on a daily basis and vary across time and context. A recent systematic review recommended standardized reporting of EMA design for studies that address weight-related behaviors.

**Objective:**

To answer the call for reporting study designs using EMA, this paper describes in detail the EMA design of the *Family Matters* study and how it was adapted over time to improve functionality and meet the needs of a racially, ethnically, and socioeconomically diverse sample.

**Methods:**

*Family Matters* is an incremental, 2-phased, mixed methods study, conducted with a racially and ethnically diverse, immigrant and refugee sample from largely low-income households, designed to examine risk and protective factors for child weight and weight-related behaviors in the home environment. The *Family Matters* study intentionally recruited White, Black, Hmong, Latino, Native American, and Somali parents with young children. Parents in phase 1 of the study completed 8 days of EMA on their smartphones, which included signal-contingent surveys (eg, asking about the parent’s stress at the time of the survey), event-contingent surveys (eg, descriptions of the meal the child ate), and end-of-day surveys (eg, overall assessment of the child’s day).

**Results:**

A detailed description of EMA strategies, protocols, and methods used in phase 1 of the *Family Matters* study is provided. Compliance with EMA surveys and participants’ time spent completing EMA surveys are presented and stratified by race and ethnicity. In addition, lessons learned while conducting phase 1 EMA are shared to document how EMA methods were improved and expanded upon for phase 2 of the *Family Matters* study.

**Conclusions:**

The results from this study provided an important next step in identifying best practices for EMA use in assessing weight-related behaviors in the home environment.

**International Registered Report Identifier (IRRID):**

DERR1-10.2196/30525

## Introduction

### Background

Ecological momentary assessment (EMA) is an innovative method used to capture real-time information about people’s health behaviors (eg, eating, physical activity, parental feeding practices, and stress and mood), which is becoming more commonly used by researchers who study weight-related behaviors. Although EMA is new to the field of weight-related health, it has been used for decades in other related fields such as smoking cessation, eating disorders, chronic pain, and sleep [1] as it is both dynamic and highly adaptable to the research questions and hypotheses being tested. EMA collects data from participants as they go about their regular lives (ecological) and involves repeated in-the-moment assessments of behaviors (momentary) [2]. In addition, EMA allows for capturing fluctuations in behaviors across time and context to identify behaviors that are more trait-like or state-like, which can be helpful in designing interventions to address weight and weight-related behaviors [3,4].

EMA was developed as a dynamic tool in response to the static nature of other self-report tools, such as retrospective surveys, which are subject to both random error and systematic bias [5]. The use of EMA across studies has varied in the frequency of assessment (eg, assessing participants hourly vs daily) and the length of the assessment (eg, days vs year-long assessment period) [2,6,7]. Furthermore, the technology used varies among studies, from paper diaries to smartphone-based apps (eg, mobile health [mHealth] technology). Studies also vary in their sampling design, whether it is time-based, event-based, or a combination [2]. EMA allows for sophisticated statistical analysis of data, such as cross-lagged models exploring how earlier events and behaviors (eg, stress) are associated with subsequent events and behaviors later on the same day or days later (eg, parent feeding practices) [3,4] and within-subject models examining how variations in the characteristics of events within a household are associated with each other (eg, preparing meals differently is associated with the types of foods served [8]).

Within the field of weight-related health behavior research, EMA is being used in the assessment of behaviors such as dietary intake and physical activity [9-16]. EMA is a valuable tool for assessing dietary intake; while eating is not a unique occurrence, the behaviors and contextual factors surrounding dietary intake (eg, parent feeding practices and family meal characteristics) fluctuate over time and are influenced by many factors, including stress, the home environment, and social interactions. EMA is well-suited for capturing fluctuations in behaviors and contextual factors and their associations with dietary intake. Similarly, EMA is being used in the field to assess behaviors and emotional states that fluctuate across time, such as mood, stress, and parental feeding practices (eg, restriction and pressure to eat) that have high potential to influence weight and weight-related behaviors. These behaviors and emotions are ideal to measure via EMA as they occur regularly on a day-to-day basis, may vary across time and context, and have a high potential to influence weight-related behaviors of children and other family members within the home environment.

A systematic review that evaluated the use of EMA to assess weight-related behaviors in youth and their families recommended standardizing the reporting of EMA measures across studies as details about EMA design are often absent and can lead to misinterpretation of study results [11]. As there is variation in the design and implementation of EMA across studies, it is important for researchers to describe their EMA protocols and processes in-depth to allow the field to develop EMA best practices going forward and to have standardization across studies to allow for the comparison of study results. In addition, as the use of EMA continues to increase in the field of weight-related health, it is important to understand how low-income, racially and ethnically diverse participants interact with data collection methods using EMA. This is especially important as children from low-income and minority households are at the highest risk for negative health outcomes (eg, obesity and cardiovascular disease) [17,18], and their households may be under higher levels of stress due to adverse environments and systemic structures that place undue burden on these populations [19]. Although several past EMA studies have included multiple races and ethnicities or targeted specific races and ethnicities, such as Latino [20] and Black or African American [21,22] participants, most EMA studies have included a predominantly White sample [12]. In addition, there appears to be a scarcity of EMA studies that specifically include immigrant populations [23].

Concerns have also been raised regarding the use of EMA with participants from low-income or low-educational attainment households and in populations who may be less technologically savvy [2]. Such populations may have jobs that make it particularly difficult to respond to EMA prompts during their work hours (eg, retail shift workers) [24]. Similarly, low-income and minority populations generally experience high levels of stress resulting from adverse environmental stimuli [25], which may make responding to EMA prompts more taxing. Therefore, understanding the application of EMA within low-income, racially and ethnically diverse, immigrant and refugee populations is the next necessary step for mHealth research.

### Objective

Given the increased use of EMA in the field of weight-related health research and its high potential for measuring important weight-related behaviors in the home environment, this study seeks to provide a detailed description of the EMA strategies, protocols, and methods used in the *Family Matters* study to investigate weight-related behaviors among participants from low-income, racially and ethnically diverse, immigrant households in order to inform other studies that aim to assess weight-related behaviors within the home environment using EMA. Lessons learned from the EMA assessment of weight-related behaviors will be shared to document how EMA methods were improved and expanded across study phases to better capture momentary behaviors and accommodate the needs of the low-income, racially and ethnically diverse, immigrant sample. The results from this study provide an important next step in identifying best practices for EMA use in assessing weight-related behaviors in the home environment.

## Methods

### Study Design and Population

*Family Matters* is an incremental, 2-phased, mixed methods study conducted with a racially and ethnically diverse, immigrant and refugee sample from largely low-income households. *Family Matters* examined risk and protective factors for child weight and weight-related behaviors in the home environment [4,16]. Phase 1 was an in-home observation of 150 families—25 each of African American, Native American, Somali, Latino, Hmong, and White families—with a child aged 5-7 years (ie, study), conducted between 2015 and 2016. Phase 2 is an ongoing longitudinal, epidemiological cohort study of racially and ethnically diverse, primarily low-income parent and child dyads (N=1307, approximately 200 families each per racial and ethnic group), with a subsample of 627 parent and child dyads who also completed the EMA. Phase 1 was intended to be both an in-depth observation of home environment risk and protective factors associated with weight and weight-related behaviors of diverse children and their families and a pilot study for our EMA protocols and analyses to inform phase 2 of our study. As phase 1 EMA informed phase 2 EMA development, we will first describe phase 1 EMA design, procedures, and protocols in detail and then discuss how it was adapted for phase 2, based on learnings from phase 1. Table 1 presents demographic characteristics of the phase 1 and phase 2 samples.

Participants from phase 1 of *Family Matters* were recruited from primary care clinics in the twin cities metro area. Families of children between the ages of 5 and 7 years (ie, the child in the study) who had a recent well-child visit were sent a recruitment letter from their primary care provider. Families were eligible if (1) the study child lived full-time with the parent or primary guardian and the child was away from home during the day (eg, school and summer camp)—to ensure all dietary and EMA measures occurred across similar contexts; (2) the child had no medical problem precluding study participation (eg, disease altering diet or physical activity, serious mental illness); (3) the child had a sibling living in the home between the ages of 2 and 12 years—as the study aims included understanding how family dynamics and structure were connected to child weight and weight-related behaviors; and (4) the family had at least 3 family meals per week. The primary study aim was to examine associations between family meal characteristics and child weight and weight-related behaviors. In addition, parents needed to be able to read and speak in English, Spanish, Hmong, or Somali.

In phase 1, mixed methods data were collected from family participants during an 8-10–day period, which included 2 home visits (parents or primary guardians were registered for EMA on an iPad mini [Apple Inc], at the first home visit, with more information to follow), and an 8-day observation period between home visits where EMA was collected. Detailed information about other measures from phase 1 collected besides EMA (eg, 24-hour dietary recalls, home food inventory, accelerometry, video-recorded task, and qualitative interviews) during in-home visits has been published elsewhere [16].

All study materials, including EMA survey questions, were translated from English into Spanish, Hmong, and Somali. The following process was used in the translation of materials: (1) a bilingual and bicultural team member translated materials into Spanish, Hmong, or Somali; (2) two additional bilingual and bicultural team members reviewed the translated materials; and (3) the 3 translators met to resolve any differences in translation, focusing on capturing the intent of the English question. The Institutional Review Board Human Subjects Committee of the University of Minnesota approved all protocols used in both phases of the *Family Matters* study.

**Table 1:** *Family Matters* phase 1 and 2 survey sample demographic characteristics (N=1457).

Participant characteristics			Primary caregiver		
			Phase 1 (n=150)		Phase 2 (n=1307)
Female, n (%)			137 (91)		1171 (90)
Age (years), mean (SD)			34.5 (7.1)		35.7 (7.9)
Born in the United States, n (%)			87 (58)		859 (66)
**Immigrant time living in the United States (years), n (%)**					
	≤1	1 (2)		8 (2)	
	1 to ≤5	5 (8)		52 (12)	
	5-10	8 (13)		51 (11)	
	≥10	48 (76)		336 (75)	
	Not reported	1 (1)		—^a^	
**Household race and ethnicity, n (%)**					
	Native American	25 (17)		211 (16)	
	Hmong	25 (17)		226 (17)	
	Black	25 (17)		280 (21)	
	White	25 (17)		239 (18)	
	Somali or Ethiopian	25 (17)		136 (10)	
	Hispanic	25 (17)		215 (16)	
**Survey language (selected by participant), n (%)**					
	English	107 (71)		1148 (88)	
	Spanish	16 (11)		134 (10)	
	Hmong	7 (5)		8 (1)	
	Somali	20 (13)		17 (1)	
**Educational attainment, n (%)**					
	Some high school	32 (21)		183 (14)	
	High school or associates	88 (59)		521 (40)	
	Some college or bachelors	11 (7)		409 (31)	
	Graduate degree	18 (12)		194 (15)	
	Not reported	1 (1)		—	
**Household income (US $), n (%)**					
	≤20,000	50 (33)		393 (30)	
	20,000-34,999	55 (37)		323 (25)	
	35,000-49,999	16 (11)		203 (16)	
	50,000-74,999	12 (8)		143 (11)	
	75,000-99,999	7 (5)		75 (6)	
	≥100,000	9 (6)		159 (12)	
	Not reported	1 (1)		11 (1)	

^a^No data were missing from phase 2.

### EMA Design and Procedures

#### Overview

For the *Family Matters* study, we used the current EMA best practice design with the following 3 types of EMA messaging [2]:

1. event-based or event-contingent messaging, where parents completed a survey after every meal occurrence they shared with the study child aged 5-7 years;
2. time-based or signal-contingent, which assessed momentary constructs (eg, stress and coping) at random intervals throughout the day;
3. end-of-day, where parents provided an overall summary of their day. All surveys were designed to be experienced by the participant as calm, soothing via a blue color scheme.

**Figure 1:**
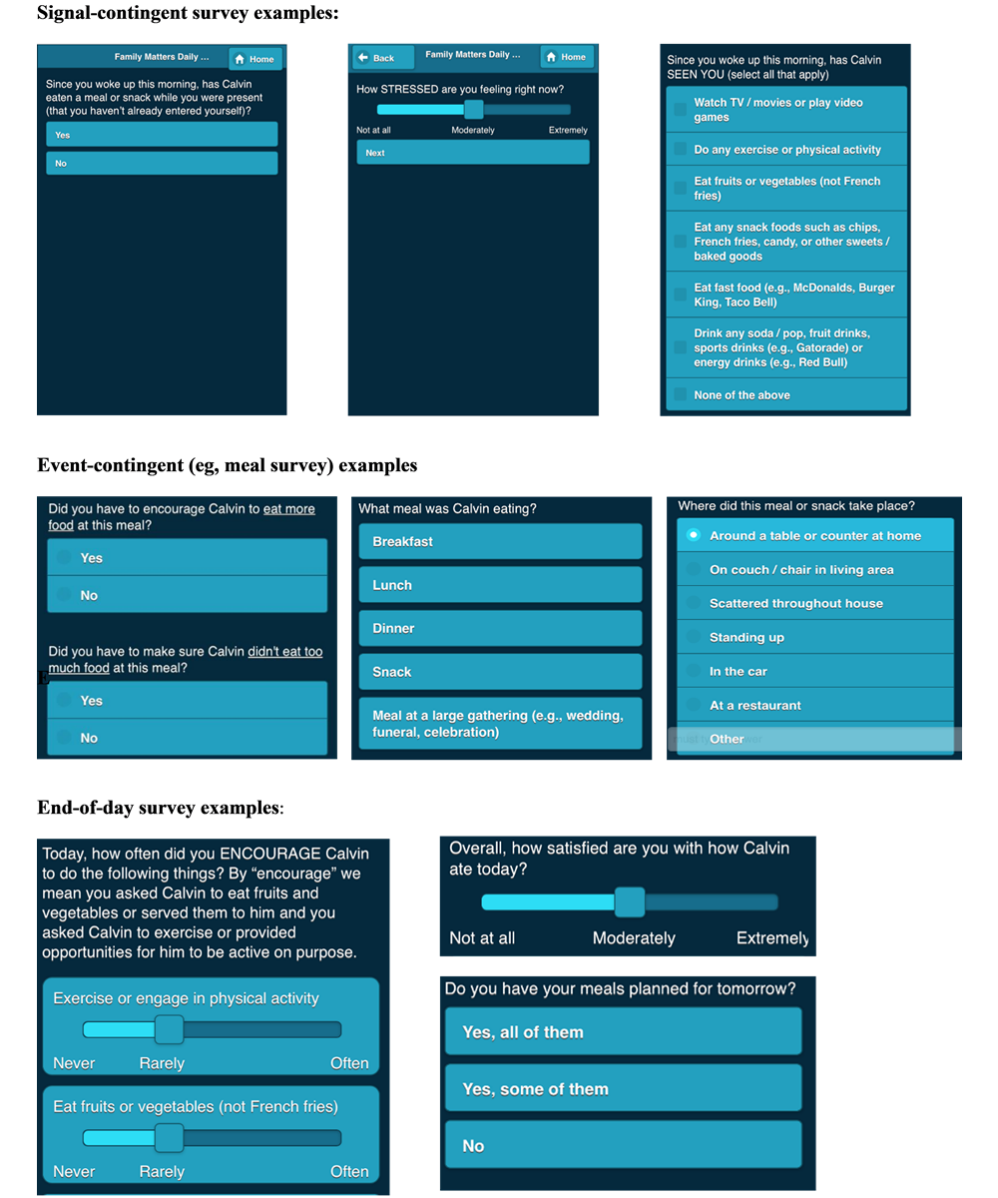
Screenshots of ecological momentary assessment survey questions answered by *Family Matters* study participants in phase 1.

Figure 1 shows examples of the color scheme used [26].

#### Registration

At the end of the first home visit, parents worked with research staff on reviewing EMA instructions and protocols and registering for EMA on a study-provided iPad mini. During the visit, research members logged into a web-based EMA registration portal developed by the *Family Matters* team and asked parents to complete the registration sections (Multimedia Appendix 1 [27-45]). Once the participant was registered, a unique survey link was created using the EMA software program. This link was then added as an icon to the iPad mini home screen. Parents clicked on this icon to complete any of their EMA surveys. The iPad mini was restricted so that participants could access the home screen icon, but other capabilities, such as purchasing apps or browsing the internet, were blocked.

A research team member reviewed a binder of EMA instructions with the parent, which was then left at home. This binder included (1) descriptions of the surveys to be completed, as well as the number of daily surveys necessary to complete; (2) screenshots reminding participants how to access EMA surveys; (3) basic information about the iPad mini, such as charging, turning the iPad mini on and off, turning up the volume (to hear survey notifications), and finding the home screen; and (4) contact information for the study team. In addition, before the home visit, the parent completed an event-contingent meal survey. This ensured that the surveys were submitted appropriately and helped introduce parents to EMA surveys. The practice surveys were excluded from the analysis. After the second home visit, the iPad mini was taken from the home and the *Family Matters* icon was removed from the home screen to ensure that other participants only used their own unique EMA survey links. Participants’ incentives to participate in all components (eg, home visits and EMA) of the *Family Matters* study included an iPad mini and the opportunity to earn up to US $100 for completing all study components.

#### Types of Surveys

##### Event-Contingent

Parents were instructed to complete an event-contingent survey (or meal survey) after every meal the study child ate when they (the parent) were present. Parents accessed the meal survey by clicking the *Family Matters* survey icon on the home screen of their iPad mini. This allowed the parent to report on meals they shared together (eg, a family meal) and on meals where only the child ate (eg, an after-school snack where both the parent and child were present). Parents were instructed not to report on meals the child ate where the parent was not present (eg, school lunch). All phase 1 EMA questions can be found in Multimedia Appendix 1. Sample visuals of EMA questions can be found in Figure 1. Survey questions were designed using validated measures and were then adapted for the EMA format. For example, instead of asking “In the last 3 months...,” the EMA question would begin, “Since you woke up this morning...” or “Since your last survey....” When appropriate, some survey questions were changed to a *yes* or *no* response format rather than a Likert scale. As shown in Figure 1, survey questions with Likert-scale response options were preset to the midpoint. Times entered by the participant (eg, wake times) were preset to the most likely AM or PM designation (eg, wake times were automatically set to AM). Other preset default response options were decided on a per-question basis by the research team. In addition, some response options were preset based on responses to earlier questions. After responding to the foods served at family meals, participants were asked which food the child ate at the meal; only the foods that were served could be selected. Similarly, the parent had to be present while the child was eating for the meal to qualify as a family meal. Therefore, when asked about who was present at the meal, the parent and the child were shown automatically as present at the meal, and the parent could add additional family members to the list. Participants had to answer all EMA questions to submit the survey; the *Next* button used to reach the next page of questions was grayed out until all questions were answered.

##### Signal-Contingent

Parents were sent 4 signal-contingent surveys per day. Signal-contingent surveys were spaced so that they began after the parent woke up (reported during the EMA registration; Multimedia Appendix 1). The time between the parents’ reported wake and sleep times was divided into 5 blocks to accommodate the 4 signal-contingent surveys and the end-of-day survey, with at least one hour separating each block (eg, a block of time from 8 to 11 AM with the next block starting at noon), so that there would never be an overlap of surveys. Scheduling signal-contingent surveys around the parent’s sleep and wake time allowed surveys to be scheduled to accommodate different life situations (eg, working an overnight shift), if needed. Parents were notified via an iPad chime when a signal-contingent survey was ready to be taken. In addition, parents could choose to have an additional prompt either texted to their phone or emailed. Parents had 1 hour to begin the signal-contingent survey on the iPad mini; if participants failed to begin the survey, the signal-contingent survey link would become inactive, although parents would still have the option to complete an event-contingent (eg, meal) survey.

In addition, for the *Family Matters* study, it was important to capture all meals the parent shared with the study child. To ensure that parents captured as many meals as possible, the first question of the signal-contingent surveys (Multimedia Appendix 1) asked the parent if they forgot to report a shared meal with the study child either after waking up or after completing the last survey. If the parent had forgotten to enter a meal survey, they were directed to complete the event-contingent survey first. After submitting the event-contingent survey, they were immediately directed back to complete the signal-contingent survey. As meals are not rare events, it was assumed that parents might forget to initiate event-contingent surveys each time they shared a meal with their child; this prompting strategy allowed the *Family Matters* study to capture additional meals that may otherwise have been forgotten.

##### End-of-Day

Parents were sent the final (fifth) survey at the start of the final scheduled block (determined by parents’ wake and sleep time). As with the signal-contingent surveys, the end-of-day survey began by asking whether the parent had shared a meal with the study child since either waking up or completing the last survey that had not yet been reported. Unlike signal-contingent surveys, which asked about in-the-moment measures (eg, How stressed are you right now?), the end-of-day survey asked for an overall assessment of the day (eg, Overall, how stressed were you today?). To help ensure that the end-of-day survey was completed, participants were given 6 hours to complete the final survey. The last question of the end-of-day survey asked the parents to assess how difficult it was for them to fill out the surveys during the day (Multimedia Appendix 1). An assessment of respondent burden is an important feature of momentary studies in that it provides information about the demographic and environmental factors that could result in periodic observation gaps or overall low compliance with the study protocol. Although this study is intensive relative to traditional cross-sectional observational studies, respondents reported that they did not experience chronic study burden or increased burden over time. On days when burden was high, participant reports of stress were elevated and burden appeared to resolve over the subsequent days, suggesting that factors other than the study instruments were related to overall burden. The strongest demographic predictor of burden was a first language other than English, suggesting that EMA protocols should anticipate and tailor data collection to diverse population needs [46].

#### Duration of EMA (Days)

All phase 1 participants (n=150) completed 8 days of EMA. The decision to include 8 days instead of 7 was based on prior research using observational methods, suggesting the need to allow for a *sensitizing period* (eg, one day) for participants to acclimate to the equipment and potentially intrusive measures [47,48]. There were also criteria established for the EMA responses to qualify as a *complete* day. Specifically, participants needed to finish (1) 2 of the 4 signal-contingent surveys, (2) at least one meal survey, and (3) the end-of-day survey. Therefore, all participants had at least 8 meal surveys and 8 end-of-day surveys. In addition, the observation window was extended if the participant was not able to complete the minimum number of surveys. For example, if the participant only completed one signal-contingent survey, an extra EMA observation day was added to their observation window until all 8 days of EMA met the criteria for a complete day. These criteria were established based on the importance of retaining participants from diverse, low-income backgrounds who sometimes cannot finish study requirements given time constraints, resources, acute stressors, or complex life situations. Having 8 full days of EMA data per participating also allowed enough power to detect differences in momentary behaviors by race and ethnicity. However, we also recognize that incomplete days may be due to the very momentary behavior the study is hoping to assess (eg, a high-stress day that limited the participant’s ability to fill out EMA prompts). Thus, both complete and incomplete days of EMA were collected, which allowed the use of both data sets as research questions and hypotheses warranted (eg, analysis of foods served at family meals may include all meal surveys regardless of whether they meet the minimum criteria to be a *complete* day).

#### Resources Used for EMA Implementation

##### Technology

A computer programmer (third author, MJ) with experience in technology-assisted research methods developed the *Family Matters* programming of EMA surveys. Our programmer had a strong understanding of web design and database architecture. For the *Family Matters* study, built-in internet information services by Microsoft were chosen because the web hosting maintenance was minimal and easy to configure. Razor syntax was chosen as the website’s programming language, and Visual Studio was chosen as the development platform for generating the .NET Razor pages. Although there are many tools available that can generate a functional survey, the chosen tools allowed for the level of complexity and control the study demanded at an affordable cost. For phase 1, SMS text message notifications were sent to participants using Twilio, along with a proprietary Windows service. For phase 2, Amazon Web Services was used in place of Twilio to meet the updated privacy policies of University of Minnesota.

##### Staff Time

Staff members were involved in phase 1 EMA in the following ways: (1) educating participants on how to use EMA and registering them in the EMA system at the first home visit, (2) tracking participants to ensure EMA surveys were completed, (3) remotely trouble shooting EMA issues with participants, and (4) deactivating participants from the EMA system at the end of their observation window.

**Figure 2:**
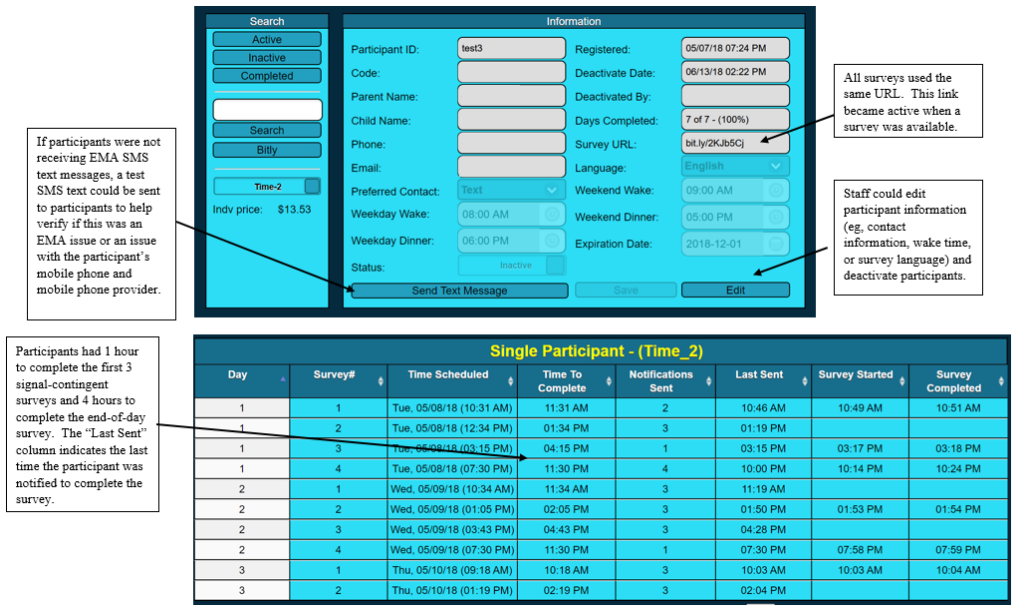
Depiction of the ecological momentary assessment tracker used in phase 2 of the *Family Matters* study. EMA: ecological momentary assessment.

To ensure that the participants were able to meet the minimum criteria for a complete EMA day and for staff to be able to identify any participant’s troubleshooting needs, our EMA programmer built a web-based tracker. The staff members were able to monitor participants’ EMA progress, including seeing when signal-contingent and end-of-day surveys were scheduled to be sent and when surveys were started and completed. Contact information for the participant, language of the surveys, and the participant’s wake and sleep times were also identified. The tracker also allowed the staff to make some changes to the EMA (eg, change the survey language) without burdening the EMA programmer. Figure 2 shows a visual illustration of the EMA tracker.

Using the EMA tracker, the staff members were instructed to contact participants: (1) if the participant did not have a *complete* first day of EMA, to ensure the participant understood the minimum requirements and to identify any troubleshooting and (2) if the participant went 2 days in a row without having a *complete* day to identify any problems or complex situations. In addition, as EMA was part of a larger study design, the staff also contacted the parent by phone during the EMA observation period in order to obtain a 24-hour dietary recall. Once the tracker indicated that the parent had completed 8 days of EMA, a staff member alerted the parent that they had successfully completed the study and deactivated their EMA.

The most common EMA difficulties were some participants’ inability to complete EMA surveys while at work, particularly as some participants were not allowed to have the iPad mini with them during their shift. In these cases, the staff worked with parents on a case-by-case basis. Most parents with difficulties completing surveys due to work were able to meet the minimum requirements (eg, they completed a signal-contingent survey before work and another after their work shift). There were a small number of parents whose work schedules did not allow for the completion of EMA (eg, a nurse working a 12-hour shift). In these rare cases, parents were asked to complete the EMA on nonwork days, and extra days were added to the observation period to ensure 8 days could be completed. Although this information was provided in the registration binder, some parents were unfamiliar with iPads or using a tablet and needed additional remote assistance (eg, how to close a survey tab and return to the home screen).

#### EMA Compliance and Analysis Considerations

##### EMA Compliance

Overall, participants completed 8 days of EMA (ie, a minimum of 2 signal-contingent surveys, 1 meal survey, and 1 end-of-day survey), an average of 10.5 days (SD 7.5 days; Multimedia Appendix 2). Somali families had the lowest average completion time (8.8, SD 3.8 days), and Black families had the highest (11.6, SD 6.2 days). Over 40.6% (61/150) of families completed all 8 days in a row without missing any days; 15.5% (23/150) of families missed 1 day and 13.5% (20/150) of families missed 2 days. Racial and ethnic differences were observed in participants’ EMA compliance; two-third (16/25, 64%) of Latino families completed EMA without missing any days, compared with only 24% (6/25) of Native American families. Black (10/25, 40%), White (8/25, 32%), and Native American (8/25, 32%) families were the racial and ethnic groups with the highest percentage of participants missing ≥4 days of EMA. The most frequent reason a participant did not fulfill requirements for a complete day was a missed signal-contingent (ie, completed <2) survey; this was true across all racial and ethnic groups. Across all racial and ethnic groups, missing a meal survey was the *least* likely reason a participant did not fulfill the requirements for a complete day. EMA surveys were intentionally kept short to ensure participant compliance. Overall, EMA surveys—regardless of whether they were signal, event, or end-of-day surveys—generally took participants less than 5 minutes to complete.

Regarding meal surveys, participants completed an average of 3.7 (SD 1.5) meal surveys on weekdays and 4.3 (SD 1.6) on weekend days. There was no variation in this pattern across the 6 racial and ethnic groups. As described earlier, participants could complete a meal survey by either (1) self-initiating the survey after sharing a meal with their child or (2) as part of the signal-contingent prompt (eg, if they forgot to submit a meal that was previously eaten). There was no difference in participants’ patterns of self-initiating meal surveys in the first half of the observation period compared with the second half (ie, participants did not stop self-initiating meal surveys once they understood that meal surveys could also be taken as part of the signal-contingent survey).

##### Analysis Considerations

For most of the analysis of phase 1 data, the data set of 1200 days (150 participants × 8 days) was used. However, there were times when using *all* EMA data—not just complete days—was appropriate. For example, a recent analysis of phase 1 EMA data investigated the concordance of foods reported in meal surveys with foods reported through 24-hour dietary recalls [49]. It was not important if the EMA meal survey was part of a *complete* day; therefore, all meal surveys in the data set were used. Future EMA data analysis should carefully consider whether restricting to only eligible days is necessary or appropriate for the study design; analyses that do not restrict should also consider whether data are missing at random.

EMA data can take both wide and long data formats, resulting in complex data management and analytical needs. As part of the data cleaning and management protocol, the analyst team developed a reference document that contained key information about how to explore the data, investigate missingness, describe panel data frequencies, and merge multiple sources of study data for analysis. The purpose of this document was to establish consistent data integrity procedures to ensure that analysts used descriptive and inferential procedures appropriate for intensive longitudinal data. Furthermore, EMA data collection often exploits *select all that apply* question formats to ensure that a comprehensive momentary assessment is captured. These question types can result in complex, hybrid data structures (a wide and long format) and are preferred to be collected in strictly long formats (ie, one participant survey per row). This minimizes the complexity of dummy coding responses at the analysis stage if the response values are stored in delimited formats. Analysts should also note that the response composition of EMA select all that apply questions will result in data values stored across many columns of data. For example, the first response that is asked can only be stored in the first dummy variable, but the last response value can be stored in any dummy variable position (if the respondent selects only the final response or if the respondent selects all response values, respectively). Study documents should be updated to describe how the raw data format generates complex data structures, and common codes to manage the data should be outlined for consistency.

#### Adapting EMA in *Family Matters* Phase 2 From Lessons Learned in Phase I EMA

##### Overview

In phase 2, 1307 diverse parent and child dyads took a web-based survey at 2 time points, approximately 18 months apart, and approximately half of these families (n=627) were also eligible for enrollment in our EMA subsample. Participants were enrolled into the study between 2016 and 2018. The EMA survey design for phase 2 differed slightly from phase 1 including the following: (1) parents were asked to complete up to 4 surveys per day, including 3 signal-contingent surveys during the day and an end-of-day survey that combined the signal-contingent questions (eg, stress level) and event-contingent meal questions specific to the family’s dinner meal; (2) parents had to complete a minimum of 2 signal-contingent and the end-of-day survey for a day to be *complete* and needed to finish 7 complete days; and (3) parents received US $75 for completing 7 days of EMA. These adaptations to our EMA protocol were directly informed by what we learned from phase 1.

##### Technology, Registration and Training, and Design Changes to Phase 2 EMA

A decision whether to use an iPad mini again or participant’s own phones was deliberated for phase 2 EMA. The main concern was whether all participants had access to a smartphone. Ultimately, we decided to use smartphones given some phase 1 participant feedback that the iPad minis were cumbersome. Thus, in phase 2, all participants chose to use their own phones, even though the *Family Matters* study had smartphones available for participants to use. Although a smartphone requirement may be restrictive for some participants, it was not restrictive for our primarily low-income (105/150, 70% of families had incomes of <US $35,000) and diverse sample. This experience aligns with findings from recent research indicating that 98% of Americans in 2020 own a mobile phone, with 81% of them being smartphones [50].

As phase 2 used participants’ smartphones, we were unable to use the same approach of placing an icon on the EMA (ie, iPad mini) device and developed a new approach. One approach considered was the development of an app, but there were many reasons why this was not ideal for our study. First, an app would be costly and time consuming to develop, and apps would have to be created for the different mobile phone operating systems used by participants (eg, iOS). In addition, there was concern that participants may have trouble or be hesitant to download the app and that it would require regular updates. Ultimately, the process for phase 2 EMA included participants being sent an SMS text message every time a survey was available, which contained the survey link that the participants followed to access the web-based survey. As there was one unique survey link per participant, participants could follow the link from any SMS text message (ie, not only the most recent one) to access EMA surveys. Participants also had the option to have survey notifications sent to their email addresses if they notified the staff that it was a better fit (eg, participants who worked primarily in front of a computer). Participants were alerted when enrolling in the study that they would receive SMS text messages and would be responsible for any SMS text message charges they incurred through their mobile phone plan.

##### Registration

Registration for phase 2 EMA was performed remotely and by the participant. After completing the web-based survey, parents who reported more than 3 family meals per week were given the opportunity to participate in an optional EMA substudy. This eligibility criteria aligned with the study aim of examining momentary mealtime routines and behaviors. Participants were able to download a form with substudy information about EMA requirements (eg, number of days and surveys needed) and interested participants were given an access code and directed to a web-based form to consent to the optional EMA substudy; they were then automatically directed to the EMA registration page. To ensure that the participants would receive texted survey links, participants were sent a test SMS text message after registration. Although staff were available to assist if necessary (and could even register participants on the web if needed), overall, participants registered themselves for EMA and understood the requirements (eg, number of surveys needed to complete) without requiring staff assistance. An important lesson learned from phase 2 of *Family Matters* is that participants—including low-income, racially and ethnically diverse, immigrant and refugee participants—are generally able to remotely enroll in an EMA study and understand the study requirements with little to no staff assistance. Depending on the overall study aims, this remote enrollment option allows for a wider geographical recruitment range and for the study to continue remotely during public health crises (eg, the COVID-19 pandemic).

##### Staff Time for Participant Tracking

Multiple features were added to the phase 2 EMA to assist both participant compliance and decrease staff time. First, multiple reminder SMS text messages were built into the signal-contingent and end-of-day survey windows. For signal-contingent surveys (expiration of 1 hour), participants received an initial SMS text message with the survey link and an SMS text message alerting the participant of the expiration time. If the survey was not completed, the participant received another reminder SMS text message after 30 minutes and another reminder SMS text message after 45 minutes for a maximum of 3 reminder SMS text messages. For end-of-day surveys (expiration of 4 hours), participants received the initial SMS text message with the survey link; they then received reminder SMS text messages every 45 minutes until the survey was complete or expired, for a maximum of 5 reminder SMS text messages.

Immediately after the end-of-day survey was completed or expired, the participant received another SMS text message with a summary of their study participation information to date, including (1) whether the participant had finished a complete day (ie, at least 2 signal-contingent and end-of-day surveys); (2) how many complete days the participant had done; and (3) if the participant had not finished a complete day, a reminder that additional observation days would be added to the EMA window to allow the participant to complete 7 days. The SMS text message also reminded the participants that they would receive US $75 after completing 7 complete days of EMA.

Regarding staff time, a feature was built into the phase 2 EMA system, where participants were automatically deactivated after 7 complete EMA days were completed. Therefore, unlike phase 1, the staff did not have to actively track each participant every day and manually deactivate. In addition, an email system was set up in which a study email account was emailed daily with the following information: (1) EMA participants who had not finished a complete EMA day on their first observation day, (2) EMA participants who had gone for more than 2 days without finishing a complete EMA day, and (3) the language of the EMA participant. This allowed the staff to easily identify the participants needing to be contacted each day to assist with any EMA difficulties or questions.

#### EMA Visual Design

As surveys were conducted on participants’ smartphones in phase 2, questions were formatted so that the participant did not have to scroll to the right or left to see the full question and response option. Similarly, pages of the survey were designed so that they contained only a small number of EMA questions, which minimized how much participants had to scroll down. Related to the smaller screen of a smartphone versus an iPad mini, the style of the response option (eg, radio button vs checkbox) was carefully considered to promote response ease. For example, questions with a Likert scale had response options provided on a slider (with anchors) rather than a pull-down menu. For the slider, the participant only had to select in the general vicinity of the anchor they wanted to choose (ie, they were not required to push a very specific section of the slider bar).

As participants were registering themselves, we provided instructions in a variety of languages (ie, English, Spanish, Somali, and Hmong) to make this possible. After completing the full web-based survey (in their preferred language), participants were directed to the EMA registration page. Instructions were provided on this page in all 4 languages, and participants were asked to enter their unique access code and then to select a survey language. For phase 2, over 93.3% (585/627) of the sample took EMA surveys in English, 6.2% (39/627) took the surveys in Spanish, and only a few families took the survey in Hmong (1/627, 0.2%) or Somali (3/627, 0.5%). Upon participant request, the staff members were able to change the language of the surveys. In addition, due to less participant and staff contact in phase 2, an information button was added to each question. Participants could select the *i* button and be provided Spanish, Hmong, and Somali translations to the survey questions and response options.

## Results

Using EMA methods in the *Family Matters* study allowed for many cutting-edge research questions to be addressed, innovative analyses to be run, and methodological approaches to be advanced. Multimedia Appendix 3 [3,4,13-15,51-55] provides a description of the selected study results highlighting the *Family Matters* research question, analysis used, principal findings, and implications for future research.

## Discussion

### Principal Findings

Overall, the main aims of this paper were to (1) answer a call in the field to report EMA study designs and (2) to extend prior EMA research by providing a detailed description of the *Family Matters* innovative EMA study design, methodology, protocols, and procedures used to investigate weight-related behaviors of children from low-income and minority households. In addition, lessons learned were also highlighted from both phases of the *Family Matters* study and are shown in Textbox 1 for future research to benefit from the development of EMA best practices and standardization of protocols across studies.

*Family Matters* demonstrated that EMA was an effective tool for collecting rich weight-related behavior data from participants from a low-income, racially and ethnically diverse, immigrant and refugee sample. In addition, phase 2 established that participants, including non–English-speaking participants, were able to register and complete EMA surveys without one-on-one or in-person staff assistance while using their personal smartphone device. Results from the *Family Matters* study will help inform future research teams using EMA, particularly around diet and physical activity, as they make decisions about the EMA study design.

**Lessons learned from the *Family Matters* study for future ecological momentary assessment (EMA) studies.**

**1. Set up and registration**

- **Lessons learned**
  - It was feasible for parent participants from low-income, racially and ethnically diverse, immigrant and refugee households (referred to in this textbox as *participants*) to successfully complete the ecological momentary assessment (EMA).
  - Participants were able to complete the EMA via their own smartphones without study provision of such devices.
  - Participants without familiarity with iPad tablet technology were able to easily learn how to operate these devices.
  - Participants preferred receiving survey notifications via SMS text message versus via email.
  - The *Family Matters* study used a computer program designed by an in-house programmer that did not rely on participants downloading an application. This also allowed the programmer to design only one system rather than multiple applications for different smartphone operating systems.
  - Participants were able to register remotely without staff assistance, which included navigating to the EMA registration page, entering an access code, and entering in registration information (eg, phone number and name). This was the case for all study participants, regardless of the main language they spoke (ie, English, Spanish, Hmong, or Somali).
  - Participants who moved into a new time zone during phase 2 needed to have their survey times adjusted, as the initial system was set up using only CST.
- **Applying lesson to future EMA studies**
  - Future studies can feel confident that EMA studies can be carried out in diverse groups, including in non–English-speaking groups.
  - Future studies may be able to rely on participants using their own smartphone devices to complete the EMA; researchers may want to consider having a small budget for providing devices to some participants who may have a mobile phone that is not a smartphone or in case of device failure.
  - Studies providing technology for participants to complete the EMA should consider providing guidance on using the technology (eg, opening web browsers and closing tabs), while at the same time feeling confident that most participants will be able to use mHealth technology.
  - Providing SMS text message notifications for the EMA is likely sufficient, although this may vary depending on the study population (eg, job primarily using a computer).
  - Although participants will become more familiar with applications, some may have hesitancy or trouble downloading an app. EMA application may be a useful tool for future research studies, although it should not feel like a requirement.
  - Although staff should be available to troubleshoot any participant questions or concerns, the *Family Matters* study demonstrated that participants were able to register for the EMA—and understand what to expect (eg, that EMA surveys would start the morning after registration)—without the staff walking them through the process.
  - Future longitudinal studies or ones where participants are located in different time zones should include a time zone question during EMA registration. The adjustment of time zones can then be read and altered by the SMS text messaging service.

**2. Tracking participants**

- **Lessons learned**
  The EMA system was set up so that (1) participants were automatically deactivated when they had finished enough complete survey days and (2) if a participant failed to finish a complete day, another day was automatically added on to their observation period.
  A protocol was designed to alert staff on when to contact participants (eg, if the participant did not finish a complete day on their first observation day). Participants received multiple reminder SMS text messages to complete each EMA survey, and participants also received a summary SMS text message at the end of each day telling them (1) if they had finished a complete day, (2) how many complete days they had done, and (3) a reminder of the incentive amount.
- **Applying lesson to future EMA studies**
  Having these study design elements automatically built into the EMA computer program significantly reduces staff time (eg, the time spent tracking and deactivating participants), and can lead to participant satisfaction and less participant confusion (eg, eliminates the possibility of sending the participant surveys to finish after the participant has completed all EMA requirements).
  Having multiple reminders to study participants increases the likelihood that participants will complete surveys. Repeated reminders also reduce the staff time needed to contact participants.

**3. EMA survey design**

- **Lessons learned**
  - EMA design for phase 2 (completed on participant smartphones) needed to consider how the survey would appear on a smartphone screen rather than how it looked on a computer screen.
  - As the EMA is a newer tool for assessing diet and physical activity The *Family Matters* study drew questions from validated surveys and altered them to be EMA-friendly (eg, changing from a Likert scale to yes or no options, changing the question heading to be *In the last month*... to *Since you woke up*...).
  - Participants were able to complete a meal survey (ie, providing information about a meal they shared with their child) in 2 ways: (1) self-initiating the survey and (2) as part of the signal-contingent prompt. Over half of meal surveys in phase 1 were completed via self-initiation. There did not appear to be a difference in participants’ patterns of self-initiating meal surveys in the first half of the observation period versus the second half.
  - Although phase 1 and 2 of *Family Matters* had criteria for what counted as a *complete* day of EMA, data from noncomplete days were kept and were valuable for many analyses (eg, analyses that focused only on meal surveys).
  - The web service used during phase 2 of the EMA needed to be changed (from Twilio to Amazon Web Services) to comply with updated privacy policies of the University of Minnesota.
- **Applying lesson to future EMA studies**
  - EMA survey design should be smartphone friendly. For example, the question text should be large enough to be viewed on a phone screen. The question should be designed so that participants do not have to scroll to read the full question or to see the response options. Questions should be on multiple pages rather than having all survey questions on only one page.
  - Researchers should consider adopting the EMA questions already being utilized in EMA survey research. Survey questions for constructs that have not been assessed via EMA should be selected using validated measures (when possible); questions and response options may need to be altered to be more EMA-friendly.
  - For researchers wanting to simplify their EMA design, assessing events (eg, smoking and eating a meal) through signaled prompts rather than participant self-initiation may be a viable option. Depending on the event being considered, researchers may want to consider adding in more signal prompts to catch more events.
  - EMA study designs should capture and retain all data submitted by participants, regardless of whether they meet the full criteria set by the researcher.
  - As the EMA will likely collect identifiable participant data (eg, phone numbers), researchers should be aware of the privacy policies of the institution they are working in to ensure they are compliant.

### Considerations for Future EMA Research

One functionality that we built into the design of our EMA event surveys was the ability of the parent to enter a meal survey at the beginning of a signal-contingent survey if they had forgotten to report a meal (event-contingent survey) earlier. This intentional design allowed us to capture any unreported meals that the parent shared with the child in the study. This design was important as EMA studies with racially and ethnically diverse, low-income households are less common; thus, we wanted to ensure that the EMA system was user-friendly to navigate and allowed to collect as much data as possible, within the bounds of the data being accurate. However, there are potential disadvantages to consider in the study design. First, it is not possible to ascertain the exact time of the behavior (ie, meal), although it is possible to determine the window of time the behavior occurred, and there could potentially be a loss of specificity further away from the meal the survey is entered. It is important that signal- and event-contingent surveys be collected in the order they occurred, so they do not affect a retrospective assessment (eg, you would not want participants to submit signal-contingent surveys, ie, report on momentary stress, but enter all event-contingent, ie, meal, surveys in the evening). Future EMA research collecting meal-level data through signal-contingent surveys may be able to increase the level of event-contingent (ie, meal survey) completion by offering multiple ways to complete the survey (ie, individually or as part of the signal-contingent survey), while also having the participant report on the time the meal was eaten when they took the delayed meal survey. Parents also only reported on meals for which both the parents and the child were present. This allowed parents to provide detailed information on the meal; however, this design may not allow for the assessment of overall child dietary intake behaviors as many meals (eg, those eaten at school) were not reported. Another important consideration for future research is related to the differing amounts of time parents are with their child. It may be important to assess the amount of time the parent spent with the child since the last survey to determine if parent behaviors could have had an influence on the child. Future research may wish to involve older children, who may be more reliable reporters than younger children of dietary intake behaviors, in EMA data collection alongside their parents.

### Conclusions

EMA is a method that was successfully implemented and improved upon in the *Family Matters* study across both study phases (ie, phase 1 and phase 2), both of which included participants from primarily low-income and racial and ethnic samples. Lessons learned about EMA study design and implementation were also shared so that researchers could benefit from them in their own future EMA studies.

## Appendix

Multimedia Appendix 1 Ecological momentary assessment questions used in phase 1 of Family Matters.

Multimedia Appendix 2 Responsiveness of phase 1 Family Matters participants to ecological momentary assessment surveys.

Multimedia Appendix 3 Selected results from the Family Matters phase 1 study using ecological momentary assessment data.

Multimedia Appendix 4 Peer-review report by the Psychosocial Risk and Disease Prevention Study Section, National Institutes of Health.

## Abbreviations

EMA: ecological momentary assessment
mHealth: mobile health
