# Supplementary material for: Ecological Momentary Assessment of Weight-Related Behaviors in the Home Environment of Children From Low-Income and Racially and Ethnically Diverse Households: Development and Usability Study
Source: JMIR Res Protoc. 2021 Dec 1;10(12):e30525. doi: 10.2196/30525 (PMC8686482; doi:10.2196/30525)
Supplement: Multimedia Appendix 1 [file resprot_v10i12e30525_app1.docx]

**Multimedia Appendix 1.** Ecological momentary assessment questions used in phase 1 of *Family Matters*.

| EMA questions | | Response options |
| --- | --- | --- |
| **Registration survey questions** | | |
|  | Language | English, Spanish, Somali, Hmong |
|  | What is your first name? | Participant wrote-in response |
|  | What is your gender? | Male , Female |
|  | What is the name of the child participating in the study? | Participant wrote in response |
|  | What is your child's gender? | Male , Female |
|  | What time do you usually get up in the morning? | Clock appears where participants can select the time. AM and PM were preset (eg, AM for morning times), although these could be changed if necessary. |
|  | What time do you usually go to bed at night | Participant filled in response |
| **Event-contingent (meal survey) questions** | | |
|  | What meal was [child’s name] eating? | Breakfast , Lunch , Dinner , Snack , Meal at a large gathering (eg, wedding, funeral, celebration) |
|  | How many children were present during the meal or snack? (Count [child’s name]) [26,27] | 1 , 2 , 3 , 4 , 5 , 6 , 7 , 8 , 9 , 10+ |
|  | Who were they? (Check all that apply) | Your child in the study [response option automatically selected] , Older sibling(s) , Younger sibling(s) , Other family members (eg, cousin) , Nonfamily members (eg, friend, neighbor) |
|  | How many adults were present during the meal or snack? (Count yourself) [26,27] | 1 , 2 , 3 , 4 , 5 , 6 , 7 , 8 , 9 , 10+ |
|  | Who were they? (Check all that apply) | Myself , Other caregiver (eg, partner, spouse) ,Other family members (eg, grandparent, aunt, uncle) , Non-family members (eg, friend, neighbor) |
|  | Who prepared this meal? [28] (Select all that apply) | Myself , My partner/spouse , A child in the household , Another adult in the household , Other person(s) (eg, pot luck, visiting a friend) , Food establishment (eg, fast food, restaurant, grocery store deli) , Other (participant provided a write-in option) |
|  | Where did this meal or snack take place? [26,27] | Around a table or counter at home , On couch/chair in living area , Scattered throughout house , Standing up , In the car , At a restaurant , Other (participant provided a write-in option) |
|  | Which best describes the type of food served? (Select all that apply) [26,27] | Fast food/take-out (eaten at home or at a restaurant) , Pre-prepared foods (eg, macaroni and cheese, frozen meals) or purchased snacks (eg, fruit snacks, chips, granola bars, cereal) , Homemade/freshly prepared (include fresh fruits or vegetables here) |
|  | Were any of the following foods served? (Select all that apply). For a dish containing multiple foods (eg, soups, sandwiches, casseroles) please select the main ingredients in the dish. For example, if you had beef and vegetable soup, mark both: *meat protein* and *vegetables*. [29,30] | Fruit , Vegetables , Whole grains (eg, whole wheat breads or cereals, brown rice, oatmeal, corn tortillas) , Refined grains (eg, white bread or cereals, flour tortillas, white rice) , Dairy (eg, milk, cheese, yogurt, milk alternate such as soy milk, ice cream) , Meat protein (eg, chicken, beef, seafood/fish) , Beans, eggs, seeds, nuts, tofu , Sugary drinks (eg, pop, Kool-Aid, Capri Sun, Sunny Delight, sports drinks) , Cake/cupcake/cookies or other baked goods , Candy (eg, sweets, chocolate, Gushers, fruit snacks) |
|  | Which of the following foods did [child’s name] eat at the meal or snack? (Select all that apply) [29,30] | *Only the foods selected above as being SERVED were included as response options. Remaining response options were grayed out.*  Fruit , Vegetables , Whole grains (eg, whole wheat breads or cereals, brown rice, oatmeal, corn tortillas) , Refined grains (eg, white bread or cereals, flour tortillas, white rice) , Dairy (eg, milk, cheese, yogurt, milk alternate such as soy milk, ice cream) , Meat protein (eg, chicken, beef, seafood/fish) , Beans, eggs, seeds, nuts, tofu , Sugary drinks (eg, pop, Kool-Aid, Capri Sun, Sunny Delight, sports drinks) , Cake/cupcake/cookies or other baked goods , Candy (eg, sweets, chocolate, Gushers, fruit snacks) |
|  | What most influenced your decision to offer these foods? (Select up to 2) [31] | Quick and easy to make , Child/family likes , Child asked for a specific food or meal , Desire to avoid conflict with child or a family fight , It was food I had available at home , It was a healthy option , Stressful day/busy schedule , Too tired to cook , It was a planned meal , It was available at the place we ate (eg, restaurant, celebration/gathering), ,Other (participant provided a write-in option) |
|  | Of the 2 answers you selected, which one influenced you most to offer these foods? | Quick and easy to make , Child/family likes , Child asked for a specific food or meal , Desire to avoid conflict with child or a family fight , It was food I had available at home , It was a healthy option , Stressful day/busy schedule , Too tired to cook , It was a planned meal , It was available at the place we ate (eg, restaurant, celebration/gathering), ,Other (participant provided a write-in option) |
|  | How long did it take to prepare this meal? [32] | No time (eg, fast food, fruit snacks or granola bar, premade, fresh fruits or vegetables, food at event/gathering) , Less than 15 minutes , 15-30 minutes , 30 minutes to 1 hour , 1 hour or more , I did not prepare it |
|  | Which of the following things were happening during the meal or snack? (Select all that apply) [33] | Conversation , Watching TV , TV on in background , Playing a video game , Using a cell phone , Using a tablet , Using a computer , Reading/looking at a book , Listening to headphones , None of the above |
|  | What was the meal or snack atmosphere like? (Select up to 2) [34] | Chaotic , Rushed , Tense , Relaxed , Enjoyable , Neutral |
|  | Of the 2 answers you selected, which describes the meal or snack atmosphere best? | Chaotic , Rushed , Tense , Relaxed , Enjoyable , Neutral |
|  | Did you have to encourage [child’s name] to eat more food at this meal? [35] | Yes , No |
|  | Did you have to make sure [child’s name] didn't eat too much food at this meal? [35] | Yes , No |
|  | What food did you have to encourage [child’s name] to eat more of? | *Only the foods selected above as being served were included as response options. Remaining response options were grayed out.*  Fruit , Vegetables , Whole grains (eg, whole wheat breads or cereals, brown rice, oatmeal, corn tortillas) , Refined grains (eg, white bread or cereals, flour tortillas, white rice) , Dairy (eg, milk, cheese, yogurt, milk alternate such as soy milk, ice cream) , Meat protein (eg, chicken, beef, seafood/fish) , Beans, eggs, seeds, nuts, tofu , Sugary drinks (eg, pop, Kool-Aid, Capri Sun, Sunny Delight, sports drinks) , Cake/cupcake/cookies or other baked goods , Candy (eg, sweets, chocolate, Gushers, fruit snacks) |
|  | What food did you have to make sure [child’s name] didn't eat too much of? | *Only the foods selected above as being served were included as response options. Remaining response options were grayed out.*  Fruit , Vegetables , Whole grains (eg, whole wheat breads or cereals, brown rice, oatmeal, corn tortillas) , Refined grains (eg, white bread or cereals, flour tortillas, white rice) , Dairy (eg, milk, cheese, yogurt, milk alternate such as soy milk, ice cream) , Meat protein (eg, chicken, beef, seafood/fish) , Beans, eggs, seeds, nuts, tofu , Sugary drinks (eg, pop, Kool-Aid, Capri Sun, Sunny Delight, sports drinks) , Cake/cupcake/cookies or other baked goods , Candy (eg, sweets, chocolate, Gushers, fruit snacks) |
|  | Did [child’s name] refuse to eat any of the food you offered him/her? [36] | Yes , No |
|  | What happened when [child’s name] refused to eat? | I made another meal or offered different food to him/her , [Child_name] ate the other foods served and not the foods he/she didn't like , [Child_name] had to taste the food and then could have a different meal option (eg, cereal, a sandwich) ,[Child_name] had to taste the food and then could leave it on his/her plate and eat from the other choices on his/her plate , [Child_name] didn't eat anything , I told [child_name] he/she couldn't have dessert if he/she didn't eat/try the food ,I made [child_name] eat the food anyway |
|  | How satisfied are you with how [child’s name] ate at this meal? | Not at all , A little , Moderately , Quite a bit , Extremely |
| **Signal-contingent survey questions** | | |
|  | Since the last survey/Since you woke up this morning, has [child’s name] eaten a meal or snack while you were present (that you haven’t already entered yourself)? | Yes , No |
|  | How many hours did [child’s name] sleep last night? [37] | *This question was asked until the participant responded. After participants provided a value, this question was no longer asked for the day.*  1 , 2 , 3 , 4 , 5, 6 , 7 , 8 , 9 , 10 , 11 , 12 |
|  | How stressed are you feeling right now? [38] | Not at all , A little , Moderately , Quite a bit , Extremely |
|  | What is the main the source of your stress? | *This question was only asked if the participant reported >Not at all stress.*  Many things to get done at home , My family makes a lot of demands from me , Conflicts or arguments with my spouse or romantic partner , Conflicts with my children or having to discipline them , A lot of work to get done at my job or school , Other (participant provided a write-in option) |
|  | Right now, how certain do you feel that you can handle all the things that you have to do today? [38] | Not at all , A little , Moderately , Quite a bit , Extremely |
|  | How sad or depressed are you feeling right now? [39] | Not at all , A little , Moderately , Quite a bit , Extremely |
|  | Since the last survey/Since you woke up this morning, how much time have you spent with [child’s name] (together in the same location)? | Not at all , Less than one hour , Between 1-2 hours , Between 3-4 hours , Between 4-5 hours , More than 5 hours |
|  | Since the last survey/Since you woke up this morning, has [child’s name] seen you (select all that apply) | Watch TV/moves or play video games , Do any exercise or physical activity , Eat fruits or vegetables (not French fries) , Eat any snack foods such as chips, French fries, candy, or other sweets/baked goods , Eat fast food (eg, McDonalds, Burger King, Taco Bell) , Drink any soda/pop, fruit drinks, sports drinks (eg, Gatorade) or energy drinks (eg, Red Bull) , None of the above |
|  | Since the last survey/Since you woke up this morning has [child's name] | Watched TV/movies or played video games , Done something physically active , Eaten any fruits or vegetables (not French fries) , Eaten any snack foods such as chips, French fries, candy, or other sweets/baked goods , Eaten any fast food (eg, McDonalds, Burger King, Taco Bell) , Drank any soda/pop, fruit drinks, sports drinks (eg, Gatorade) or energy drinks (eg, Red Bull) , None of the above |
|  | Are you and [child’s name] wearing your activity monitor right now? | Yes , No |
| **End-of-day survey questions** | | |
|  | Since the last survey/Since you woke up this morning, has [child’s name] eaten a meal or snack while you were present (that you haven’t already entered yourself)? | Yes , No |
|  | Overall, how stressful was your day? [38] | Not at all , A little , Moderately , Quite a bit , Extremely |
|  | Overall, how certain did you feel that you could cope with all the things that you had to do today? [38] | Not at all , A little , Moderately , Quite a bit , Extremely |
|  | Overall, what caused you the most stress today? | A lot of work at home , A lot of work at school or job , A lot of demands from my family , Financial problems , Conflicts/arguments with a spouse or romantic partner , Conflicts or disciplinary problems with my children , Other (participant provided a write-in option) |
|  | Did any of the following things happen as a result of being stressed today? | I fixed an easy or quick meal , I bought fast food for a meal , Everyone made their own meal ,We skipped a meal , None of the above |
|  | Overall, how sad or depressed did you feel today? [39] | Not at all , A little , Moderately , Quite a bit , Extremely |
|  | Overall, how much time have you spent with [child’s name] today (together in the same location)? | Not at all , Less than one hour , Between 1-3 hours , Between 4-6 hours , Between 7-9 hours , More than 10 hours |
| **Today, how often did (child's name) see you:** | | |
|  | Watch TV/ movies or play video games [40] | Never , Rarely , Moderately , Often |
|  | Exercise or engage in physical activity [41] | Never , Rarely , Moderately , Often |
|  | Eat fruits or vegetables (not French fries) [42] | Never , Rarely , Moderately , Often |
|  | Eat snack foods such as chips, French fries, candy, or other sweets / baked goods [42] | Never , Rarely , Moderately , Often |
|  | Eat fast food (eg, McDonalds, Burger King, Taco Bell) [43] | Never , Rarely , Moderately , Often |
|  | Drink soda / pop, fruit drinks, sports drinks (eg, Gatorade) or energy drinks (eg, Red Bull) [42] | Never , Rarely , Moderately , Often |
|  | Today, how often DID [CHILD'S NAME]: | Never , Rarely , Moderately , Often |
|  | Watch TV/ movies or play video games [40] | Never , Rarely , Moderately , Often |
|  | Exercise or engage in physical activity [41] | Never , Rarely , Moderately , Often |
|  | Eat fruits or vegetables (not French fries) [42] | Never , Rarely , Moderately , Often |
|  | Eat snack foods such as chips, French fries, candy, or other sweets / baked goods [42] | Never , Rarely , Moderately , Often |
|  | Eat fast food (eg, McDonalds, Burger King, Taco Bell) [43] | Never , Rarely , Moderately , Often |
|  | Drink soda / pop, fruit drinks, sports drinks (eg, Gatorade) or energy drinks (eg, Red Bull) [42] | Never , Rarely , Moderately , Often |
|  | Today, how often did you LIMIT the following things for [child's name]? By *limit* we mean you did not let [child's name] do these things on purpose. Limiting also includes not having these foods or a TV in your home on purpose so [child's name] can’t have access to them. | Never , Rarely , Moderately , Often |
|  | Limited intake of snack foods such as chips, French fries, candy, or other sweets / baked goods [35] | Never , Rarely , Moderately , Often |
|  | Limited intake of fast food (eg, McDonalds, Burger King, Taco Bell) [35] | Never , Rarely , Moderately , Often |
|  | Limited intake of soda / pop, fruit drinks, sports drinks (eg, Gatorade) or energy drinks (eg, Red Bull) [35] | Never , Rarely , Moderately , Often |
|  | Limited TV / movie or video game time [44] | Never , Rarely , Moderately , Often |
|  | Today, how often did you encourage [CHILD'S NAME] to do the following things? By *encourage* we mean you asked Calvin to eat fruits and vegetables or served them to him and you asked [child's name] to exercise or provided opportunities for him/her to be active on purpose. | Never , Rarely , Moderately , Often |
|  | Exercise or engage in physical activity [32,44] | Never , Rarely , Moderately , Often |
|  | Eat fruits or vegetables (not French fries) [35] | Never , Rarely , Moderately , Often |
|  | Do you have your meals planned for tomorrow? | Yes, all of them , Yes, some of them , No |
|  | Overall, how often did you have to encourage [child’s name] to eat more food today? [35] | Never , Rarely , Moderately , Often |
|  | Overall, how often did you have to make sure [child’s name] didn't eat too much food today? [35] | Never , Rarely , Moderately , Often |
|  | Overall, how satisfied are you with how [child’s name] ate today? | Not at all , A little , Moderately , Quite a bit , Extremely |
|  | Overall, how difficult was it for you to fill out the surveys today? | Not at all , A little , Moderately , Quite a bit , Extremely |
