# Supplementary material for: Ecological Momentary Assessment of Weight-Related Behaviors in the Home Environment of Children From Low-Income and Racially and Ethnically Diverse Households: Development and Usability Study
Source: JMIR Res Protoc. 2021 Dec 1;10(12):e30525. doi: 10.2196/30525 (PMC8686482; doi:10.2196/30525)
Supplement: Multimedia Appendix 2 [file resprot_v10i12e30525_app2.docx]

Multimedia Appendix 2. Responsiveness of phase 1 *Family Matters* participants to ecological momentary assessment EMA surveys.

|  | **Full Sample**  **N=150** | | **Black**  **n=25** | | **White**  **n=25** | | **Hmong**  **N=25** | |
| --- | --- | --- | --- | --- | --- | --- | --- | --- |
| **Response Characteristic** | Weekday | Weekend | Weekday | Weekend | Weekday | Weekend | Weekday | Weekend |
| Average Completion Time (Days) | 10.5, SD: 7.5 | | 11.6, SD: 6.2 | | 11.4, SD: 10.4 | | 10.4, SD: 10.2 | |
| Average Missed Days | 3.5, SD: 7.5 | | 4.6, SD: 6.2 | | 4.4, SD: 10.4 | | 3.4, SD: 10.2 | |
|  |  |  |  |  |  |  |  |  |
| Survey Completion Time (Minutes) |  |  |  |  |  |  |  |  |
| Signal-Contingent Surveys | 2.2, SD: 33.0 | 1.5, SD: 6.4 | 3.5, SD: 43.4 | 1.0, SD: 0.9 | 1.2, SD: 1.5 | 1.2, SD: 1.5 | 4.5, SD: 71.6 | 1.1, SD: 1.1 |
| Event-Contingent (i.e., Mealtime) Surveys | 4.3, SD: 41.6 | 2.8, SD: 2.0 | 2.5, SD: 2.2 | 2.2, SD: 1.4 | 4.8, SD: 52.0 | 2.2, SD: 1.1 | 6.4, SD: 84.2 | 2.5, SD: 1.8 |
| End of Day Survey | 5.1, SD: 41.4 | 3.2, SD: 3.8 | 7.7, SD: 66.2 | 3.3, SD: 7.5 | 2.8, SD: 1.8 | 3.1, SD: 2.2 | 2.6, SD: 2.0 | 2.5, SD: 2.3 |
|  |  |  |  |  |  |  |  |  |
| Frequency of Missed Days per Participant |  |  |  |  |  |  |  |  |
| No Missed Days | 61 (41.2%) | | 9 (36%) | | 10 (40%) | | 7 (28%) | |
| 1 Day | 23 (15.5%) | | 4 (16%) | | 2 (8%) | | 8 (32%) | |
| 2 Days | 20 (13.5%) | | 2 (8%) | | 3 (12%) | | 4 (16%) | |
| 3 Days | 8 (5.4%) | | 0 (0%) | | 2 (8%) | | 3 (12%) | |
| 4+ Days | 36 (24.3%) | | 10 (40%) | | 8 (32%) | | 3 (12%) | |
|  |  |  |  |  |  |  |  |  |
| Time to First Missed Survey | 4.3, SD: 1.9 | | 3.8, SD: 1.6 | | 4.7, SD: 1.8 | | 4.8, SD: 2.4 | |
|  |  |  |  |  |  |  |  |  |
| Average Meal Surveys per Day | 3.7, SD: 1.5 | 4.3, SD: 1.6 | 3.5, SD: 1.6 | 4.5, SD: 2.0 | 3.4, SD: 1.2 | 3.7, SD: 1.2 | 3.8, SD: 1.4 | 4.6, SD: 1.7 |
|  |  |  |  |  |  |  |  |  |
| Average Event Contingent Meal Surveys per Day | 2.1, SD: 1.0 | 2.5, SD: 1.3 | 2.0, SD: 1.0 | 2.6, SD: 1.7 | 2.0, SD: 0.8 | 2.5, SD: 1.1 | 1.8, SD: 0.8 | 2.4, SD: 1.6 |
| Average Signal Contingent Daily Surveys per Day | 3.5, SD: 0.7 | 3.3, SD: 0.8 | 3.4, SD: 0.7 | 3.4, SD: 0.8 | 3.6, SD: 0.7 | 3.4, SD: 0.7 | 3.3, SD: 0.7 | 3.3, SD: 0.8 |
|  |  |  |  |  |  |  |  |  |
| Reason for Noncompliance Frequencies |  |  |  |  |  |  |  |  |
| Missed Daily Survey | 527/1248 (42.2%) | | 109/268 (40.7%) | | 56/158 (35.4%) | | 127/301 (42.2%) | |
| Missed Meal Survey | 74/1248 (6%) | | 15/268 (6%) | | 16/158 (10%) | | 24/301 (8%) | |
| Missed End of Day Survey | 307/1248 (24.6%) | | 59/268 (22%) | | 53/158 (34%) | | 91/301 (30%) | |
| Missed for Multiple Reasons | 340/1248 (27.2%) | | 85/268 (31.7%) | | 33/158 (21%) | | 59/301 (20%) | |
|  |  |  |  |  |  |  |  |  |
| Frequency of Self-Initiating Mealtime Surveys |  |  |  |  |  |  |  |  |
| Early (Front Half of Observation) | 665/1888 (35.2%) | 665/1855 (35.8%) | 665/1495 (44.5%) | 665/1519 (43.8%) | 253/656 (38.6%) | 253/643 (39.3%) | 316/1002 (31.5%) | 316/896 (35.3%) |
| Late (Back Half of Observation) | 426/1379 (30.9%) | 426/1311 (32.5%) | 426/1285 (33.1%) | 426/1233 (34.6%) | 236/610 (38.7%) | 236/541 (43.6%) | 265/803 (33%) | 265/850 (31.2%) |

|  | **Hispanic**  **n=25** | | **Native American**  **n=25** | | **Somali**  **n=25** | |
| --- | --- | --- | --- | --- | --- | --- |
| **Response Characteristic** | Weekday | Weekend | Weekday | Weekend | Weekday | Weekend |
| Average Completion Time (Days) | 10.3, SD: 8.1 | | 10.2, SD: 3.6 | | 8.8, SD: 3.8 | |
| Average Missed Days | 3.3, SD: 8.1 | | 3.2, SD: 3.6 | | 1.8, SD: 3.8 | |
|  |  |  |  |  |  |  |
| Survey Completion Time (Minutes) |  |  |  |  |  |  |
| Signal-Contingent Surveys | 1.7, SD: 1.4 | 1.9, SD: 1.6 | 1.0, SD: 1.1 | 2.4, SD: 15.7 | 1.8, SD: 1.7 | 1.5, SD: 1.2 |
| Event-Contingent (i.e., Mealtime) Surveys | 4.8, SD: 18.1 | 3.7, SD: 2.6 | 2.3, SD: 1.5 | 2.4, SD: 1.6 | 4.7, SD: 14.9 | 3.5, SD: 2.7 |
| End of Day Survey | 5.2, SD: 3.3 | 4.9, SD: 3.1 | 2.7, SD: 1.7 | 2.6, SD: 2.1 | 9.7, SD: 76.5 | 2.8, SD: 2.7 |
|  |  |  |  |  |  |  |
| Frequency of Missed Days per Participant |  |  |  |  |  |  |
| No Missed Days | 16 (67%) | | 6 (24%) | | 13 (54%) | |
| 1 Day | 0 (0%) | | 5 (20%) | | 4 (17%) | |
| 2 Days | 2 (8%) | | 5 (20%) | | 4 (17%) | |
| 3 Days | 2 (8%) | | 1 (4%) | | 0 (0%) | |
| 4+ Days | 4 (17%) | | 8 (32%) | | 3 (13%) | |
|  |  |  |  |  |  |  |
| Time to First Missed Survey | 3.7, SD: 2.1 | | 4.0, SD: 1.6 | | 4.3, SD: 2.0 | |
|  |  |  |  |  |  |  |
| Average Meal Surveys per Day | 3.9, SD: 1.9 | 4.6, SD: 1.5 | 3.4, SD: 1.6 | 4.1, SD: 1.5 | 3.8, SD: 1.5 | 3.9, SD: 1.3 |
|  |  |  |  |  |  |  |
| Average Event Contingent Meal Surveys per Day | 2.4, SD: 1.4 | 2.5, SD: 0.9 | 2.2, SD: 1.0 | 2.7, SD: 1.2 | 1.8, SD: 0.9 | 1.5, SD: 0.7 |
| Average Signal Contingent Daily Surveys per Day | 3.5, SD: 0.7 | 3.3, SD: 0.7 | 3.4, SD: 0.7 | 3.1, SD: 0.7 | 3.4, SD: 0.7 | 3.3, SD: 0.8 |
|  |  |  |  |  |  |  |
| Reason for Noncompliance Frequencies |  |  |  |  |  |  |
| Missed Daily Survey | 64/172 (37.2%) | | 114/222 (51.4%) | | 57/127 (44.9%) | |
| Missed Meal Survey | 10/172 (5.8%) | | 4/222 (1.8%) | | 5/127 (3.9%) | |
| Missed End of Day Survey | 48/172 (27.9%) | | 41/222 (18.5%) | | 15/127 (11.8%) | |
| Missed for Multiple Reasons | 50/172 (29.1%) | | 63/222 (28.4%) | | 50/127 (39.4%) | |
|  |  |  |  |  |  |  |
| Frequency of Self-Initiating Mealtime Surveys |  |  |  |  |  |  |
| Early (Front Half of Observation) | 295/41 (39.8%) | 295/867 (34%) | 243/684 (35.5%) | 343/547 (44.4%) | 180/850 (21.2%) | 171/979 (17.5%) |
| Late (Back Half of Observation) | 345/1089 (31.7%) | 345/893 (38.6%) | 307/869 (35.3%) | 307/924 (33.2%) | 180/1203 (15%) | 180/1445 (12.5%) |
