# Supplementary material for: Ecological Momentary Assessment of Weight-Related Behaviors in the Home Environment of Children From Low-Income and Racially and Ethnically Diverse Households: Development and Usability Study
Source: JMIR Res Protoc. 2021 Dec 1;10(12):e30525. doi: 10.2196/30525 (PMC8686482; doi:10.2196/30525)
Supplement: Multimedia Appendix 3 [file resprot_v10i12e30525_app3.docx]

Multimedia Appendix 3. Selected results from the *Family Matters* Phase I study using ecological momentary assessment data

| EMA-related research question | Quantitative analysis used | Main results | Implications for future research |
| --- | --- | --- | --- |
| Examination of the association between transient and chronic stressors and food-related parenting practices [3] | Time-lagged approach | - Transient stressors (eg, interpersonal conflicts) were significantly associated with less healthy parenting practices (eg, restrictive feeding practices, serving fast food) that same evening. Chronic stressors (eg, recent divorce) were not consistently associated with less healthy parenting practices. | - Interventions focused on promoting healthy food-related practices should educate parents on the role transient stressors may play in their food-related parenting behaviors. - Ecological Momentary Intervention (EMI) should be considered in future interventions as a way to intervene on transient stressors experienced by parents and promote more healthful food-related parenting practices. |
| Examination of the association between momentary factors (eg, stress) with food-related parenting practices later in the day [4] | Time-lagged approach | - Parents experiencing increased stress and depressed mood earlier in the day was associated with less healthy parenting practices (eg, pressure to eat, serving less homemade meals) that same evening. Effect modification found differences by racial and ethnic group. | - Health care professionals may want to educate parents at well-child visits about the associations between parents’ experience of stress and depressed mood with less healthy feeding behaviors. - Researchers may want to consider using EMI in future interventions as a way to target momentary factors (eg, stress) and promote more healthful food-related parenting practices. |
| Description of the breakfast, lunch, and dinner meal characteristics and the meal characteristics associated with family meal frequency [50] | Multiple-linear regression | - Some consistent patterns were found across weekend and weekday family meals (eg, homemade food most commonly reported, meals took place around table, most meals included conversation). - Significant and negative associations were found between some meal characteristics (eg, serving fast food) and family meal frequency. | - Study results can be utilized in future interventions to increase family meal frequency. |
| Examination of the differences in meal characteristics (eg, location, use of media devices, meal atmosphere) between shared meals and snack occasions [51] | Cross-tabulations for panel data and generalized estimating equations (GEE) | - There were significant differences between shared meal characteristics and snacking occasions (eg, compared with meals, parents were less likely to prepare snacks, snacks were less likely to be homemade, and snacks were more likely to be eaten on the couch.). Screen time-only activities at snacks were more common for children who were overweight compared with those who were not overweight. | - Study results provide information for future interventions wanting to improve children’s snacks—the actual food served as well as the environment in which the snack is consumed. |
| Examination of the association between momentary factors (eg, stress), parent feeding behaviors, and child eating behaviors in food insecure and food secure homes [4] | Time-lagged approach | - Momentary factors (eg, parental stress) earlier in the day were associated with different parent feeding and child eating behaviors later in the day depending on the household’s food security status; parents in food insecure households experiencing high levels of stress used more restrictive feeding practices and served more preprepared foods compared with parents in food secure households. | - Health care professional should consider screening parents for food insecurity as well as momentary factors (stress, depressed mood) and providing education on the association between these factors and unhealthful feeding behaviors. - EMA should be considered in future interventions to target parental stress, and thereby, improve food-related parenting practices. |
| Mixed methods exploration of meal characteristics and parent feeding practices during family meals in food insecure and food secure homes [52] | Multiple regression, Qualitative analysis | - Many meal characteristics were significantly associated with a family being food insecure (eg, how the meal was prepared, the meal location, foods served). | - Study results provide guidance for future interventions wanting to improve family meal practices and parent feeding practices, particularly in families with limited resources. |
| Examining both the variability of, and the predictors of, parent feeding practices [53] | Cross-tabulations for panel data and multi-level models | - Parent feeding practices were more variable across the week than stable, indicating that feeding practices may be more state-like than trait-like. Parents using pressure-to-eat feeding practices were more likely to use these practices in the middle of the week compared to weekend days. Over half (69%) and nearly half (49%) of parents used pressure to eat and restrictive (respectively) feeding practices across the week. - Several meal characteristics (eg, meal type, meal setting, and emotional atmosphere) were significantly associated with parent feeding practices at meal occasions. | - Future interventions should consider using EMI to target predictors of parent feeding practices. - Health care providers can provide education to parents regarding reducing the use of restrictive and pressure-to-eat feeding practices, including the meal characteristics that may promote these parent feeding practices. |
| Examining whether home-cooked meals vary compared to pre-prepared meals in the presence of nutritious ingredients [13] | Within-participant (fixed effects) methods | - Fully or partially home-cooked family meals (eg, included fresh fruits of vegetables with a frozen pizza) were more likely to contain vegetables and fruit than those meals that were pre-prepared. | - Future interventions may want to encourage families to include more fresh foods or home-cooked foods into their family meals. |
| Comparison of parent-reported child dietary intake data collected through EMA compared with 24-hour dietary recalls [14] | Concordance analysis (eg, interrater reliability measures) | - Concordance between EMA dietary intake data and 24-hour dietary recalls was highest for breakfast and snacks. Concordance of specific foods ranged from moderately high agreement (eg, meat), moderate agreement (eg, sweets and fruit), and fair-low agreement (refined grains). Concordance was highest if the meals reported occurred at home. | - Concordance was reasonably good for dietary intake data collected via EMA. Collection of dietary intake via EMA may be an acceptable alternative to the collection of 24-hour dietary recalls. |
| Comparison of parent-reported child physical activity collected through EMA compared with accelerometry [12] | Generalized estimating equations (GEE) | - There were strong associations between EMA-reported physical activity and accelerometry data for sedentary, light, and moderate to vigorous physical activity. Some of these associations were stronger during the weekends compared with weekdays. | - Collection of child physical activity via EMA may be an acceptable alternative to the collection of accelerometry data. |
| Examination of the association between sources of stress with family meal decisions and child health behaviors [54] | Cross-tabulations for panel data and generalized estimating equations (GEE) | - Common meal-related decisions in response to stress included fixing an easy meal, everyone fixing their own meal, and having fast food for the family meal. Parents with many family demands were more likely to report their child eating an unhealthy snack. | - Future interventions may want to target parental stress as well as support parents in their planning of family meals. |
