# Supplementary material for: Ecological Momentary Assessment of Weight-Related Behaviors in the Home Environment of Children From Low-Income and Racially and Ethnically Diverse Households: Development and Usability Study
Source: JMIR Res Protoc. 2021 Dec 1;10(12):e30525. doi: 10.2196/30525 (PMC8686482; doi:10.2196/30525)
Supplement: Multimedia Appendix 4 [file resprot_v10i12e30525_app4.pdf]

SUMMARY STATEMENT
(Privileged Communication)

PROGRAM CONTACT:
Charlotte Pratt
(301) 435-0382
prattc@nhlbi.nih.gov

Release Date: **06/09/2014**

Application Number: **1 R01 HL126171-01A1**
Formerly: **1R01HD079427-01A1**

Principal Investigator

BERGE, JERICA M PHD

Applicant Organization: UNIVERSITY OF MINNESOTA

Review Group: **PRDP**
Psychosocial Risk and Disease Prevention Study Section

Meeting Date: **06/02/2014**
Council: **OCT 2014**
Requested Start: **09/01/2014**
RFA/PA: **PA13-302**
PCC: **HHCG N**

Project Title: **Addressing Unanswered Questions About the Home Environment and Childhood Obesity Disparities**
SRG Action: **Impact Score: 16 Percentile: 6**
Next Steps: **Visit http://grants.nih.gov/grants/next_steps.htm**
Human Subjects: 30-Human subjects involved - Certified, no SRG concerns
Animal Subjects: 10-No live vertebrate animals involved for competing appl.
Gender: **1A-Both genders, scientifically acceptable**
Minority: **1A-Minorities and non-minorities, scientifically acceptable**
Children: **1A-Both Children and Adults, scientifically acceptable**
Clinical Research - not NIH-defined Phase III Trial

Project Year	Direct Costs Requested	Estimated Total Cost
1	499,792	711,366
2	499,976	711,628
3	499,759	711,319
4	499,089	710,365
5	499,966	711,613
TOTAL	2,498,582	3,556,291

ADMINISTRATIVE BUDGET NOTE: The budget shown is the requested budget and has not been adjusted to reflect any recommendations made by reviewers. If an award is planned, the costs will be calculated by Institute grants management staff based on the recommendations outlined below in the COMMITTEE BUDGET RECOMMENDATIONS section.

EARLY STAGE INVESTIGATOR, NEW INVESTIGATOR

1R01HL126171-01A1 Berge, Jerica

EARLY STAGE INVESTIGATOR
NEW INVESTIGATOR

RESUME AND SUMMARY OF DISCUSSION: This application requests support to conduct a mixed methods study to investigate interpersonal and food aspects of the home environment that influence childhood obesity. This resubmitted work was very responsive to previous critique comments, and now offers exceptional high impact work that concerns the pressing public health problem of childhood obesity and additionally addresses health disparities. Reviewers again noted many strengths: the outstanding significance of understanding familial and home environment causes of childhood obesity in socioeconomic and racially/ethnic diverse families; the novel look at role of multiple caregivers and the home interpersonal environmental risk and protective factors relying on observational ecologic momentary assessment; the exceptional investigators and research environment well poised to conduct this work; and the rigorous two phased incremental mixed methods approach which includes measurement of parent stress, sibling interaction, a community based participatory approach, accelerometers and child qualitative interviews; an outstanding recruitment plan and finally an exceptional and sophisticated analytic plan as well. Reviewers noted a few minor questions: concern that the study is sufficiently powered to look at racial differences; and possible insufficient cultural tailoring of study assessments that may limit the validity of the findings. Overall reviewers expressed very high enthusiasm for this work that offers high promise to impact childhood obesity and inform future childhood obesity interventions.

DESCRIPTION (provided by applicant): While the prevalence of childhood obesity may have started to plateau for some groups of children, other groups such as low-income and minority children are experiencing increases in childhood obesity. These growing disparities may be linked to unanswered questions regarding the home environment and childhood obesity. For example, research has shown that healthful food availability/accessibility in the home, frequent family meals, and authoritative parenting style are associated with healthful dietary intake, better psychosocial health, and fewer unhealthy weight control behaviors in youth, but findings are inconsistent across studies with minority and low-income families. These conflicting findings suggest that mixed- methods studies are needed for an in-depth examination of the home environments of diverse families to identify potential explanatory mechanisms of childhood obesity that may have been overlooked in prior research. In addition, previous studies have not included state-of-the-art measures that may help illuminate factors in the home environment that differ by race/ethnicity. The primary objective of this study is to identify how familial factors, including interpersonal relationships that exist between family members, of racially/ethnically and socioeconomically diverse children act as risk or protective factors for predicting childhood obesity. To achieve this objective, a two-phased incremental mixed-methods approach will be used. Phase I (yrs. 1-2) will include in-home observations of diverse families (n=120; 20 each of African American, American Indian, Hispanic, Hmong, Somali and white families) to identify individual, dyadic (i.e., parent/child; siblings), and familial factors that are associated with, or moderate associations with, childhood obesity. The in-home observations, using our community-based participatory research partners, will include: (1) an interactive observational family task and family interview; (2) ecological momentary assessment (EMA) 17 of parent stress, mood and parenting practices; and (3) child accelerometry and 24-hour dietary recalls. Using state-of-the-art measures, such as EMA, will allow for identifying within-day fluctuations in parenting practices or parent stress levels, which may help to identify nuances within the home environment that amplify or exacerbate childhood obesity risk. Results from the in-home observations will be used for rich analyses and to inform the development of a culturally-appropriate survey in Phase II (yrs. 3- 5). The survey will be administered at two time points to a diverse sample of up to two caregivers (n=2400) of children ages 5-7. Individual, dyadic, and familial factors that are longitudinally associated with child BMIz score and weight-related behaviors will be identified. Phase I and II recruitment will occur via the electronic

Primary Care Research Network in Minnesota primary care clinics (n=82). This comprehensive evaluation of diverse home environments will identify potential factors that increase childhood obesity risk in order to create culturally-tailored interventions that will be effective in reducing childhood obesity disparities.

PUBLIC HEALTH RELEVANCE: This two-phased incremental mixed-methods study will address unanswered questions related to the home environment and childhood obesity disparities. Phase I (n=120 diverse families) includes in-home observations to gain an in-depth understanding of aspects of the home environment that are relevant to childhood obesity. Phase II (n=2400 diverse caregivers) examines longitudinal risk and protective factors for childhood obesity.

CRITIQUE 1:

Significance: 2
Investigator(s): 1
Innovation: 1
Approach: 2
Environment: 1

Overall Impact: The overarching goals of this study are to identify how familial factors play a role in childhood obesity. This highly skilled research team, led by an Early Stage and New Investigator, proposes a two phase study. During phase 1, they will conduct a qualitative analysis of the in-home environments of 120 families (20 of each race/ethnicity). The in-home assessment will include observing an interactive family task (mainly to desensitize the families to a subsequent interview), followed by a video-recorded interview with the parent and the child, use of ecological momentary assessment for one week to assess parental stress, mood and parenting practices, placement of an accelerometer on the child, and a 24-hour dietary recall. Additional 24-hour recalls will be collected, with a final one collected at the second home visit during which the accelerometers will be collected. Using data from phase 1, they will create a survey and administer it to 2400 parents (400 of each race/ethnicity) of children ages 5-7 years old (2 parents per household) at two points in time, 18 months apart (thus allowing for longitudinal data analyses). All participants will be recruited through a Primary Care Research network in Minnesota primary care clinics (n=82). As outlined below, there are many aspects of this study that are quite significant and innovative. Their approach is comprehensive and rigorous; few concerns were identified.

1. Significance:

Strengths

- Obesity is a significant public health problem and is most pronounced among low-income families and racially/ethnically diverse families. This study proposes to examine the causes of childhood obesity in these priorities populations.
- Families play an important role in children’s risk for obesity.
- The researchers intend to recruit a very racially/ethnically diverse sample including African-Americans, Hmong, Hispanics, Caucasians, and American Indians. They have specified that most of the Hispanics are of Mexican origin and the African population is Somali.
- A better understanding of the home food environment (social and physical) will help inform future interventions.

Weaknesses

- Results about the Hispanic sample may not generalize to other Latino subgroups. (minor)

- Concerns remain regarding the overlap in their research questions compared with other funded research. One would assume that some of what they will learn in Phase 1 they will have learned from the R21. (minor)

2. Investigator(s):

Strengths

- Led by a talented early stage and new investigator, Dr. Jerica Berge, this is an excellent team with expertise in all of the needed areas.
- Dr. Berge has a PhD is trained in behavioral medicine and has a specific expertise in family science. She has other research that is similar including having completed a K12 related to weight and eating.
- Dr. Neumark-Sztainer has expertise in eating behaviors in children.
- Dr. Crow provides expertise in EMA.
- Dr. Miner provides expertise in statistics.
- They have modified their research team to streamline the process and include additional expertise in health disparities research.

Weaknesses

- This remains a fairly large team and may present a challenge for this new investigator. (minor)

3. Innovation:

Strengths

- The examination of multiple caregivers' parenting styles is innovative. And examining their concordance/discordance in parenting styles on child's risk for obesity is novel.
- Very little research exists on differences in parenting styles among such a diverse group of parents as is proposed in the current study.
- The examination of parenting stress, mood and practices over a course of a week and how that is related to children's weight and weight related behaviors is novel.
- The in-home task is novel.

Weaknesses

- No concerns

4. Approach:

Strengths

- This is a mixed methods study that involves observations, ecological momentary assessment, and collection of measured data and questionnaires. They provided additional justification for Phase 1 which is a major strength of this study as it is allow us to peer into the homes of these families to explore novel aspect of the home environment to target in future interventions.
- Their observational component will examine sibling interactions.
- Data will be collected from children and multiple family members thus providing a more comprehensive picture of the home environment than what is typically obtained
- Excellent theoretical approach.

- A survey validation study is embedded between Phase 1 and 2.
- The constructs they will measure in Phase 2 is very comprehensive.
- The use of NDS 24-hour dietary recalls and accelerometers is a strength.
- Most of the methods they employ have been pilot tested. And they have excellent response rates in these previous studies.
- Their involvement of the SoLaHmo members will help maximize their recruitment and data collection efforts.
- Their focus on the 5-7 years old is excellent as it represents an important developmental period.
- Their analytic approach is well-conceived, sophisticated and articulates well with the aims.
- A major strength of this revised application is a second measurement time-point during Phase 2, thus allowing for longitudinal data analysis and prediction of behaviors and health outcomes.

Weaknesses

- Minor concerns remain about their ability to recruit fathers.

5. Environment:

Strengths

- The academic environment at the University of Minnesota is excellent.
- Their partnership with the Primary Care Research Network will support feasibility of recruitment.

Weaknesses

- No concerns.

Protections for Human Subjects:

Acceptable Risks and/or Adequate Protections

- No concerns; excellent description of how they will minimize risks.

Data and Safety Monitoring Plan (Applicable for Clinical Trials Only):

  - Although not a clinical trial, the researchers have created a plan that will minimize risks to participants.

Inclusion of Women, Minorities and Children:

G1A - Both Genders, Acceptable

M1A - Minority and Non-minority, Acceptable

C1A - Children and Adults, Acceptable

- 120 racially/ethnically diverse families will be recruited to participate in phase 1. 2400 racially/ethnically diverse parents/caregivers and one child per household (5-7 years old) will be recruited to participate in Phase 2.

Vertebrate Animals:

Not Applicable (No Vertebrate Animals)

Biohazards:

Not Applicable (No Biohazards)

Resubmission:

- The researchers addressed most of the concerns raised by the reviewers including: discussing the representativeness of the sample; their decision to assess for differences by race/ethnicity (and they are powered to do so); they will control for parent/caregiver weight status and they will stratify their analyses by child weight; their inclusion criteria for number of family members and contact with child; their CBPR approach is appropriate; no concerns with work-related restrictions on using the IPAD; additional information provided on the accelerometer data collection.

Budget and Period of Support:

Recommend as Requested

CRITIQUE 2:

Significance: 2
Investigator(s): 1
Innovation: 1
Approach: 3
Environment: 1

Overall Impact: We have insufficient data detailing how the home environment is associated with obesity risk among children in racial/ethnic minority and low income households. The present investigation aims to use a two-phase mixed methods design to assess associations of familial factors with obesity risk among children. Phase 1 will involve assessment of the home environment of children (ages 5-7) in a diverse community. Phase 1 will culminate with the development of a "culturally sensitive" survey that will be administered in Phase 2 to a diverse sample of two caregivers, twice over a 2 year period. The applicants have been responsive to the prior set of reviews, particularly in modifying the investigation into a prospective trial. Several very minor methodological concerns remain, regarding the choice of measures and characterization of the sample.

1. Significance:

Strengths

- Having a more nuanced understanding of the links between the home environment and childhood obesity may inform the development of innovative interventions.
- Familial factors play an important role in impacting childhood obesity and this study will contribute to our understanding of these processes in high risk populations.
- The quantitative aims in Phase 1 seem to extend much of the existing literature to racial/ethnic minority familiars who are at higher obesity risk.
- Examining the multiple family caregivers is a strength given difference in the composition of racial/ethnic minority households.

Weaknesses

- None noted.

2. Investigator(s):

Strengths

- The Principal Investigator is an accomplished new investigator, who is supported by a strong team of experienced co-investigators.
- There appears to be requisite experience in the investigator team for an ambitious study of this type.

Weaknesses

- None noted.

3. Innovation:

Strengths

- Examining variability in parental factors and the association with childhood behaviors.
- Combined evaluation of familial factors along with those of the home environment.

Weaknesses

- None noted.

4. Approach:

Strengths

- Theoretical model is sound and well suited to the study
- The team has strong preliminary evidence lending support to the feasibility of the proposed methods.
- Although CBPR isn't absolutely necessary for this study, that the investigators have good community relationships might enhance their likelihood of recruitment success.
- Training plan for community and research data collectors is sound.

Weaknesses

- It is unclear whether there is sufficient power to detect difference between racial/ethnic groups, as Phase II power analyses are focused on within-group differences.
- It is unclear how choice of family task (specifically, the familiarity of the chosen scenario) will affect participant responses.
- Translation and checking for cultural appropriateness is likely insufficient to ensure the validity of the measures utilized to measure child health behaviors.
- It is unclear how African American is being defined and whether Black immigrants will be included in this group.

5. Environment:

Strengths

- This is an excellent environment for the proposed study.

Weaknesses

- None noted.

Protections for Human Subjects:

Acceptable Risks and/or Adequate Protections

Data and Safety Monitoring Plan (Applicable for Clinical Trials Only):

- Acceptable

Inclusion of Women, Minorities and Children:

G1A – Both Genders, Acceptable

M1A – Minority and Non-minority, Acceptable

C1A – Children and Adults, Acceptable

Vertebrate Animals:

Not Applicable (No Vertebrate Animals)

Biohazards:

Not Applicable (No Biohazards)

Budget and Period of Support:

Recommend as Requested

CRITIQUE 3:

Significance: 2
Investigator(s): 1
Innovation: 3
Approach: 2
Environment: 1

Overall Impact: This study aims to explore familial factors, including interpersonal relationships that exist between family members of racially/ethnically and socioeconomically (SES) diverse children and how these may act as risk or protective factors for predicting childhood obesity. The investigators were mostly responsive to previous critiques. This study is significant for a variety of reasons given below. The investigators have the expertise and previous collaboration experience to carry out the study. Concerns still remain with regard to innovation and the narrow range of the population of children (e.g., 5 to 7 year olds) in the proposal. The environment is excellent.

1. Significance:

Strengths

- Investigators were mainly responsive to previous critiques.
- Factors related to obesity among children are multifaceted, and the home food environment is an important setting to examine these issues.
- Given that racial and ethnic minorities in the U.S. are disproportionately burdened by obesity, this study will contribute greatly to the understanding of why these disparities exist.

- Strong measures are proposed including accelerometer, 24-hour dietary recalls and BMI z-scores.
- Inclusion of multiple family members and family structure will help contribute to the understanding of the breadth of familial factors that may influence child dietary and physical activity behaviors.

Weaknesses

- While the power analysis informs a sample size that should be adequate for detecting differences among groups, the inclusion of such diverse racial/ethnic minority groups, as opposed to choosing a more narrow racial or ethnic group(s) (e.g. Hispanics only, but considering subpopulations based on country of origin), may limit generalizability of the overall findings (e.g., diluting the results).

2. Investigator(s):

Strengths

- The investigators have strong background and the necessary experience to conduct the study.
- Dr. Berge is a promising early stage and new investigator. Her previous research is within relevant content areas (e.g., childhood obesity, CBPR as a methodology, and working with ethnic minority populations and youth).
- The research team has extensive collaborations which will help support this project.
- Dr. Berge has several publications relevant to this study (e.g., eating behaviors, structural and interpersonal characteristics of family meals, and family home eating and physical activity environments).

Weaknesses

- None noted.

3. Innovation:

Strengths

- Variables of parental stress, mood, and practices measured throughout the day will provide new and important aspects to the home food and PA environments, especially among children.
- As the proposal states, little is known about whether parenting style and concordance or discordance differs by race/ethnicity. This study will add greatly to this understanding.

Weaknesses

- In the proposed study, it is expected that the majority of Hispanics will be Mexican; however, as previous reviewers stated, and as research has shown, obesity risks and behaviors vary among Hispanic subgroups. By potentially aggregating non-Mexican American and Mexican American families, important differences among these Hispanic subgroups could be masked.
- One of the prior reviewers stated concern as to many of the constructs in the survey development phase being already evaluated and linked to obesity. The investigators were only mildly responsive to this original concern since they state in their response that they will look for additional measures if needed but did not actually provide measures that might be novel and/or important. For instance, as stated below, measurement of food insecurity may have been helpful to incorporate (especially since the investigators briefly mentioned the construct).
- The proposal briefly mentions examining food security, but doesn’t go into adequate detail regarding how it will be measured or analyzed. It would be expected that food security status,

especially among racial/ethnic minorities, would interact with measures of parental mood, stress, and feeding practices.

4. Approach:

Strengths

- The utilization of mixed methods approach is a key strength.
- Strong approach and measures incorporated (e.g., CBPR, EMA, accelerometry, and multiple 24-hr dietary recalls) are notable strengths.
- Recruitment goals will likely be met, as sampling from 82 clinics, coupled with utilization of the Electronic Primary Care Research Network, will provide a multitude of opportunities to access the target populations.
- The incremental approach outlined by the investigators will help facilitate capturing both the qualitative and quantitative of potential risk or protective factors for childhood obesity in diverse families.
- The use of the Family Systems Theory will provide direction and assist with synthesis of findings.
- Stratified sampling to ensure inclusion of overweight and healthy weight youth will ensure the strength and robustness of the data.

Weaknesses

- Although the investigators responded to the previous critique regarding the age of the children in the proposal (5-7 year olds), this was not adequately addressed. Although potentially under-researched, this narrow age range of 5-7 years of age may result in findings that may be difficult to interpret. For instance, would the results be an effect of age or race/ethnicity?

5. Environment:

Strengths

- The investigators have adequate office space for staff, equipment, supplies, and data housing, as well as appropriate technology (secure network, software) to carry out the study.
- The Family Medicine Clinics have excellent access to the racial and ethnic minority populations of interest.

Weaknesses

- None observed.

Protections for Human Subjects:

Acceptable Risks and/or Adequate Protections

Data and Safety Monitoring Plan (Applicable for Clinical Trials Only):

Acceptable

Inclusion of Women, Minorities and Children:

G1A - Both Genders, Acceptable

M1A - Minority and Non-minority, Acceptable

C1A - Children and Adults, Acceptable

Vertebrate Animals:

Not Applicable (No Vertebrate Animals)

Biohazards:

Not Applicable (No Biohazards)

Resubmission:

- The investigators were mainly responsive to previous critiques.

Budget and Period of Support:

Recommend as Requested

THE FOLLOWING RESUME SECTIONS WERE PREPARED BY THE SCIENTIFIC REVIEW OFFICER TO SUMMARIZE THE OUTCOME OF DISCUSSIONS OF THE REVIEW COMMITTEE ON THE FOLLOWING ISSUES:

PROTECTION OF HUMAN SUBJECTS (Resume): ACCEPTABLE. There are no concerns.

INCLUSION OF WOMEN PLAN (Resume): ACCEPTABLE. Both genders will be included in the study sample.

INCLUSION OF MINORITIES PLAN (Resume): ACCEPTABLE. All race and ethnic groups will be eligible and targeted for enrollment.

INCLUSION OF CHILDREN PLAN (Resume): ACCEPTABLE. Children aged 5-7 and adult caregivers will be enrolled in this study.

NIH has modified its policy regarding the receipt of resubmissions (amended applications). See Guide Notice NOT-OD-10-080 at http://grants.nih.gov/grants/guide/notice-files/NOT-OD-10-080.html.
The impact/priority score is calculated after discussion of an application by averaging the overall scores (1-9) given by all voting reviewers on the committee and multiplying by 10. The criterion scores are submitted prior to the meeting by the individual reviewers assigned to an application, and are not discussed specifically at the review meeting or calculated into the overall impact score. Some applications also receive a percentile ranking. For details on the review process, see http://grants.nih.gov/grants/peer_review_process.htm#scoring.

MEETING ROSTER

Psychosocial Risk and Disease Prevention Study Section
Risk, Prevention and Health Behavior Integrated Review Group
CENTER FOR SCIENTIFIC REVIEW
PRDP
June 02, 2014 - June 03, 2014

CHAIRPERSON

EPSTEIN, LEONARD H, PHD
SUNY DISTINGUISHED PROFESSOR
DEPARTMENT OF PEDIATRICS
DIVISION OF BEHAVIORAL MEDICINE
STATE UNIVERSITY OF NEW YORK AT BUFFALO
BUFFALO, NY 14214

MEMBERS

ABRANTES, ANA M, PHD
ASSOCIATE PROFESSOR
DEPARTMENT OF PSYCHIATRY AND HUMAN BEHAVIOR
ALPERT MEDICAL SCHOOL
BUTLER HOSPITAL
BROWN UNIVERSITY
PROVIDENCE, RI 02906

AYALA, GUADALUPE X, MPH, PHD
PROFESSOR
INSTITUTE FOR BEHAVIORAL
AND COMMUNITY HEALTH
SAN DIEGO STATE UNIVERSITY
SAN DIEGO, CA 92123

BASKIN, MONICA L, PHD
PROFESSOR
DIVISION OF PREVENTIVE MEDICINE
UNIVERSITY OF ALABAMA AT BIRMINGHAM
BIRMINGHAM, AL 35294

BEETS, MICHAEL W, PHD *
ASSOCIATE PROFESSOR
DEPARTMENT OF EXERCISE SCIENCE
ARNOLD SCHOOL OF PUBLIC HEALTH
UNIVERSITY OF SOUTH CAROLINA
COLUMBIA, SC 29208

BEFORT, CHRISTIE , PHD *
ASSOCIATE PROFESSOR
CO-DIRECTOR, BREAST CANCER SURVIVORSHIP
CENTER
DEPARTMENT OF PREVENTIVE MEDICINE
AND PUBLIC HEALTH
UNIVERSITY OF KANSAS MEDICAL CENTER
KANSAS CITY, KS 66160

BENNETT, GARY G, PHD
PROFESSOR OF PSYCHOLOGY
DEPARTMENT OF GLOBAL HEALTH AND MEDICINE
DUKE UNIVERSITY
DURHAM, NC 27705

BOUTELLE, KERRI N, PHD
PROFESSOR
DEPARTMENT OF PEDIATRICS
AND PSYCHIATRY
UNIVERSITY OF CALIFORNIA, SAN DIEGO
LA JOLLA, CA 92037

COUPS, ELLIOT J, PHD
ASSOCIATE PROFESSOR
DEPARTMENT OF MEDICINE
RUTGERS, THE STATE UNIVERSITY OF NEW JERSEY
ROBERT WOOD JOHNSON MEDICAL SCHOOL
NEW BRUNSWICK, NJ 08901

DAVISON, KIRSTEN , PHD
ASSOCIATE PROFESSOR
DEPARTMENT OF NUTRITION
HARVARD SCHOOL OF PUBLIC HEALTH
BOSTON, MA 02115

FOCHT, BRIAN CARL, PHD *
ASSOCIATE PROFESSOR
KINESIOLOGY, DEPARTMENT OF HUMAN SIENCES
COMPREHENSIVE CANCER CENTER
AND SOLOVE RESEARCH INSTITUTE
THE OHIO STATE UNIVERSITY
COLUMBUS, OH 43210

LEAHEY, TRICIA M, PHD
ASSISTANT PROFESSOR
WEIGHT CONTROL AND DIABETES
RESEARCH CENTER
MIRIAM HOSPITAL
BROWN MEDICAL SCHOOL
PROVIDENCE, RI 02903

LEVINE, MICHELE D, PHD
ASSOCIATE PROFESSOR
DEPARTMENT OF PSYCHIATRY
UNIVERSITY OF PITTSBURGH
PITTSBURGH, PA 15213

PAUL, IAN M, MD *
PROFESSOR
DEPARTMENT OF PEDIATRICS
COLLEGE OF MEDICINE
PENNSYLVANIA STATE UNIVERSITY
HERSHEY, PA 17033

POLLAK, KATHRYN I, PHD
ASSOCIATE PROFESSOR
DUKE CANCER CONTROL
AND POPULATION SCIENCES
DUKE UNIVERSITY MEDICAL CENTER
DURHAM, NC 27705

QUARELLS, RAKALE COLLINS, PHD
ASSOCIATE PROFESSOR
CARDIOVASCULAR RESEARCH INSTITUTE
MOREHOUSE SCHOOL OF MEDICINE
ATLANTA, GA 30310

SEVICK, MARY A, SCD, BSN *
PROFESSOR OF MEDICINE
DEPARTMENT OF POPULATION HEALTH
SCHOOL OF MEDICINE
NEW YORK UNIVERSITY
NEW YORK, NY 10016

STAIANO, AMANDA E, PHD *
ASSISTANT PROFESSOR
LABORATORY OF PEDIATRIC
OBESITY AND HEALTH BEHAVIOR
PENNINGTON BIOMEDICAL RESEARCH CENTER
LOUSIANA STATE UNVERSITY
BATON ROUGE , LA 70808

TATE, DEBORAH F, PHD
ASSOCIATE PROFESSOR
DEPARTMENT OF HEALTH
BEHAVIOR AND NUTRITION
UNIVERSITY OF NORTH CAROLINA
CHAPEL HILL, NC 27599

THOMPSON, DEBORAH I, PHD
ASSOCIATE PROFESSOR OF PEDIATRICS
USDA/ARS SCIENTIST/NUTRITIONIST-CHILD OBESITY
CHILDREN'S NUTRITION RESEARCH CENTER
BAYLOR COLLEGE OF MEDICINE
HOUSTON, TX 77030

WOLIN, KATHLEEN Y, SCD *
ASSOCIATE PROFESSOR
DEPARTMENTS OF PUBLIC HEALTH
SCIENCES AND SURGERY
STRITCH SCHOOL OF MEDICINE
LOYOLA UNIVERSITY CHICAGO
MAYWOOD, IL 60153

YAROCH, AMY L, PHD
EXECUTIVE DIRECTOR
GRETCHEN SWANSON CENTER
FOR NUTRITION
OMAHA, NE 68114

MAIL REVIEWER(S)

SCHUR, ELLEN A, MD
ASSISTANT PROFESSOR
DIVISION OF INTERNAL MEDICINE
DEPARTMENT OF MEDICINE
UNIVERSITY OF WASHINGTON
SEATTLE, WA 98104

SMITH, MICHAEL T, PHD
PROFESSOR
DEPARTMENT OF PSYCHIATRY, AND BEHAVIORAL
SLEEP
MEDICINE PROGRAM, CENTER FOR BEHAVIOR
AND HEALTH, SCHOOL OF MEDICINE
BAYVIEW MEDICAL CENTER JOHNS HOPKINS
UNIVERSITY
BALTIMORE, MD 21224

SCIENTIFIC REVIEW ADMINISTRATOR

FITZSIMMONS, STACEY , MPH, PHD
SCIENTIFIC REVIEW OFFICER
CENTER FOR SCIENTIFIC REVIEW
NATIONAL INSTITUTES OF HEALTH
BETHESDA, MD 20892

GRANTS TECHNICAL ASSISTANT

JENNINGS, CHRISOULA , BS
LEAD EXTRAMURAL SUPPORT
CENTER FOR SCIENTIFIC REVIEW
NATIONAL INSTITUTES OF HEALTH
BETHESDA, MD 20892

* Temporary Member. For grant applications, temporary members may participate in the entire meeting or may review only selected applications as needed.

Consultants are required to absent themselves from the room during the review of any application if their presence would constitute or appear to constitute a conflict of interest.
